# A membrane lipid signature unravels the dynamic landscape of group 1 innate lymphoid cells across the health-disease continuum

**DOI:** 10.1016/j.isci.2025.112043

**Published:** 2025-02-17

**Authors:** Halle C. Frey, Xin Sun, Fatima Oudeif, Darleny L. Corona, Zijun He, Taejoon Won, Tracy L. Schultz, Vern B. Carruthers, Amale Laouar, Yasmina Laouar

**Affiliations:** 1 Department of Microbiology and Immunology, University of Michigan Medical School, Ann Arbor, MI 48109, USA; 2 Graduate Program of Microbiology and Immunology, University of Michigan Medical School, Ann Arbor, MI 48109, USA; 3 Department of Biomedical and Translational Sciences, Carle Illinois College of Medicine, University of Illinois Urbana-Champaign, Urbana, IL 61801, USA; 4 Child Health Institute of New Jersey, Robert Wood Johnson Medical School-Rutgers University, New Brunswick, NJ 08901, USA

**Keywords:** Lipid, Immunology, Components of the immune system, Cell biology

## Abstract

In an era where established lines between cell identities are blurred by intra-lineage plasticity, distinguishing stable from transitional states is critical, especially within Group 1 ILCs, where similarity and plasticity between NK cells and ILC1s obscure their unique contributions to immunity. This study leverages AsGM1—a membrane lipid associated with cytotoxic attributes absent in ILC1s—as a definitive criterion to discriminate between these cell types. Employing this glycosphingolipid signature, we achieved precise delineation of Group 1 ILC diversity across tissues. This lipid signature captured the binary classification of NK and ILC1 during acute liver injury and remained stable when tested in established models of NK-to-ILC1 plasticity driven by TGFβ or Toxoplasma gondii. The detection of AsGM1 at the iNK stage, prior to Eomes expression, and its persistence in known transitional states, positions AsGM1 as a pivotal marker for tracing NK-to-ILC1 transitions, effectively transcending the ambiguity inherent to the NK-to-ILC1 continuum.

## Highlights

• Glycolipid AsGM1 discriminates NK cells from ILC1s across multiple tissues
• AsGM1^+^ and AsGM1^-^ Group 1 ILCs are transcriptomic counterparts of NK cells and ILC1
• Glycolipid AsGM1 marks NK-to-ILC1 continuum driven by TGFβ in the salivary glands
• Glycolipid AsGM1 captures high dynamics within Group 1 ILCs in *T. gondii* infection

## Graphical abstract

**Figure:**
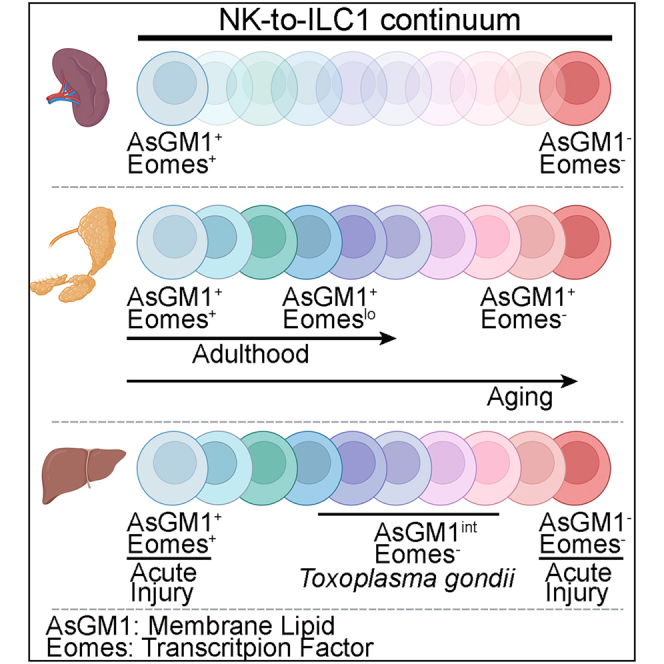

## Introduction

Innate lymphoid cells (ILCs) represent a dynamically evolving group of immune cells, comprising multiple lineages that play a crucial role in orchestrating rapid responses to environmental stressors, microbial agents, and the complex cytokine microenvironm-ent.^1^^,^^2^ This broad activation mode positions ILCs as indispensable sentinels, finely tuned to react and adapt to environmental fluctuations.^3^^,^^4^^,^^5^^,^^6^ Recent advances from transcriptomic and epigenomic studies provided insights into how ILCs may adapt to these fluctuations, suggesting that intra-lineage plasticity may play an important role in this process.^7^^,^^8^ This adaptability, evident across both human^9^^,^^10^^,^^11^^,^^12^^,^^13^^,^^14^ and murine^15^^,^^16^^,^^17^^,^^18^^,^^19^^,^^20^^,^^21^^,^^22^^,^^23^ studies, introduces a significant layer of complexity to ILC classification, challenging the assignment of specific identities and roles. This challenge is particularly acute in the Group 1 ILC lineage, where significant plasticity and extensive overlapping traits between Natural Killer (NK) cells and Type 1 ILCs (ILC1s) obscure their precise identification, particularly in infection and cancer.^18^^,^^19^^,^^20^^,^^21^^,^^22^^,^^23^^,^^24^

The task of distinguishing ILC1s from NK cells relies on a suite of markers—Eomes and CD49b for NK cells, contrasted with CD200R, CD127, CD103, CD49a, and TRAIL for ILC1s depending on the anatomical site.^25^^,^^26^^,^^27^^,^^28^ However, the efficacy of these markers in delineating ILC1s from NK cells is challenged by their variable expression. First, the presence or absence of Eomes alone may not be sufficient to consistently distinguish NK cells from ILC1s due to its variable expression during NK cell activation.^29^ Additionally, the low or absent Eomes expression observed in a subset of circulating human NK cells^30^ suggests that its reliability as a distinguishing marker may be limited. Second, CD200R can be upregulated on liver NK cells^20^ and is less effective in humans due to its expression in both ILC1s and NK cells.^31^ Third, CD103 is inducible under the influence of TGFβ but is not expressed on most ILC1 subsets.^18^ Fourth, the expression of CD127 on immature NK cells^32^ (absent in mature NK cells) and its inconstant expression on ILC1s across tissues^33^ (e.g., absent in salivary gland ILC1s) makes this marker unreliable for distinguishing between NK cells and ILC1s. Fifth, the property of TRAIL as a selective marker for liver ILC1s is undermined by its inducible expression on NK cells in response to inflammatory cytokines.^34^^,^^35^ Lastly, the upregulation of CD49a and down-regulation of CD49b on NK cells upon murine cytomegalovirus infection and cytokine stimulation highlights the challenges these markers present in identifying NK and ILC1 subsets.^36^^,^^37^^,^^38^ Consequently, the current immunological framework lacks stable markers that can unambiguously distinguish ILC1s from NK cells across health and disease states. Moreover, recent data from fate mapping mouse models, adoptive transfers, and transcriptomic profiling in diverse biological contexts including endocrine tissues,^18^ breast and prostate tumors,^28^ and *Toxoplasma gondii* (*T. gondii*) infection,^22^ identified a spectrum of intermediate subsets within the NK-to-ILC1 continuum that introduced an additional layer of complexity to the precise delineation of the Group 1 ILC landscape.^18^^,^^19^^,^^20^^,^^21^^,^^22^^,^^23^^,^^24^ Defined by unique combinations of markers specific to either NK cells or ILC1s, the identification of these transitional states—known as ILC1-like cells—has disrupted the binary classification of ILC1s and NK cells, further complicating cell identity assignment within the Group 1 ILC landscape.

The quest for the precise segregation between ILC1s and NK cells stems from a key functional divergence^36^^,^^39^: the inherent cytotoxic capabilities of NK cells, crucial for the immune system’s primary defense against infections and tumors, are absent in ILC1s. In addressing this challenge, our strategy capitalized on the unique functional specificity of Asialo-GM1 (AsGM1)—a neutral membrane glycolipid^40^^,^^41^^,^^42^ known for its long-standing association with cytotoxic activities.^43^^,^^44^^,^^45^^,^^46^^,^^47^ Through comprehensive analysis specific to each Group 1 ILC subset, complemented by transcriptomic profiling and the use of well-established models known to foster intra-lineage plasticity, the strategic use of AsGM1 has not only enabled a precise demarcation of NK cells from their non-cytotoxic ILC1 counterparts across diverse anatomical sites, but also unveiled, in environments conducive to NK-to-ILC1 plasticity, a gradient of expression that captured a previously underappreciated level of diversity within the Group 1 ILC lineage. Our strategic focus on AsGM1, a membrane lipid marker empirically linked to cytotoxic activities, thus offers a unified approach to elucidate the diversity and dynamics of Group 1 ILCs, transcending the ambiguity inherent to the NK-to-ILC1 continuum.

## Results

### AsGM1 strategically segregates group 1 innate lymphoid cells into ILC1 and natural killer cells across tissues

**Figure 1:**
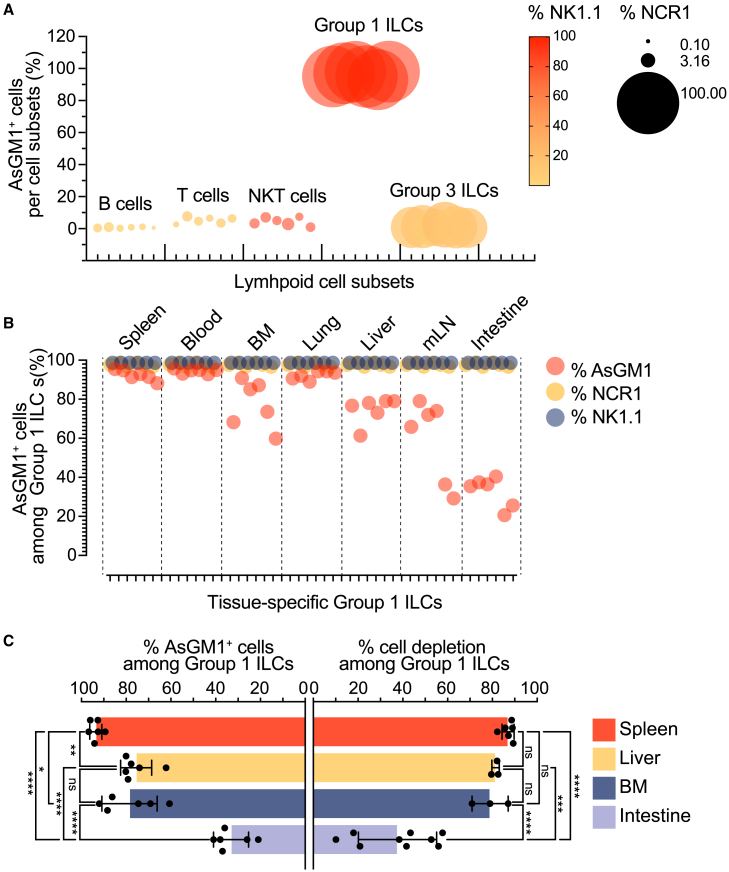
AsGM1 expression is a Hallmark of the Group 1 ILC compartment (A) Frequency of AsGM1^+^ cells among immune cells: splenic B cells (CD45^+^CD19^+^), T cells (CD45^+^CD3^+^), NKT cells (CD45^+^CD3^+^NK1.1^+^), Group 1 ILCs (CD45^+^CD3^−^CD19^−^RORγt^-^NK1.1^+^NCR1^+^), and intestinal Group 3 ILCs (CD45^+^CD3^−^CD19^−^RORγt^+^). A color gradient indicates the frequency of NK1.1^+^ cells, and bubble size represents the frequency of NCR1^+^ within each cell subset. (B) Frequency of AsGM1^+^ cells (red) in Group 1 ILCs across various tissues: spleen, blood, bone marrow (BM), lung, liver, mesenteric lymph nodes (mLN), and intestine (lamina propria). Co-expression of NCR1 (yellow) and NK1.1 (blue) is displayed as controls. (C) Mice were left untreated or treated with anti-AsGM1 antibody on days 0 and 2 and analyzed on day 3. Left: Frequency of AsGM1+ cells among Group 1 ILCs (CD45^+^CD3^−^CD19^−^RORγt^-^NK1.1^+^NCR1^+^) in the spleen, liver, bone marrow, and small intestine lamina propria. Right: Percentage of cell depletion by anti-AsGM1 treatment within Group 1 ILCs across the indicated tissues, calculated as: 100 – [(% Group 1 ILCs after treatment/% Group 1 ILCs before treatment) × 100]. Data represent 2–3 independent experiments with *n* = 3–9. Statistical analysis was performed using one-way ANOVA (C), with significance indicated by ∗*p* < 0.05, ∗∗*p* < 0.01, ∗∗∗*p* < 0.001, ∗∗∗∗*p* < 0.0001. Error bars represent mean +s.d. Refer to Figure S1 and S2 for additional information.

**Figure 2:**
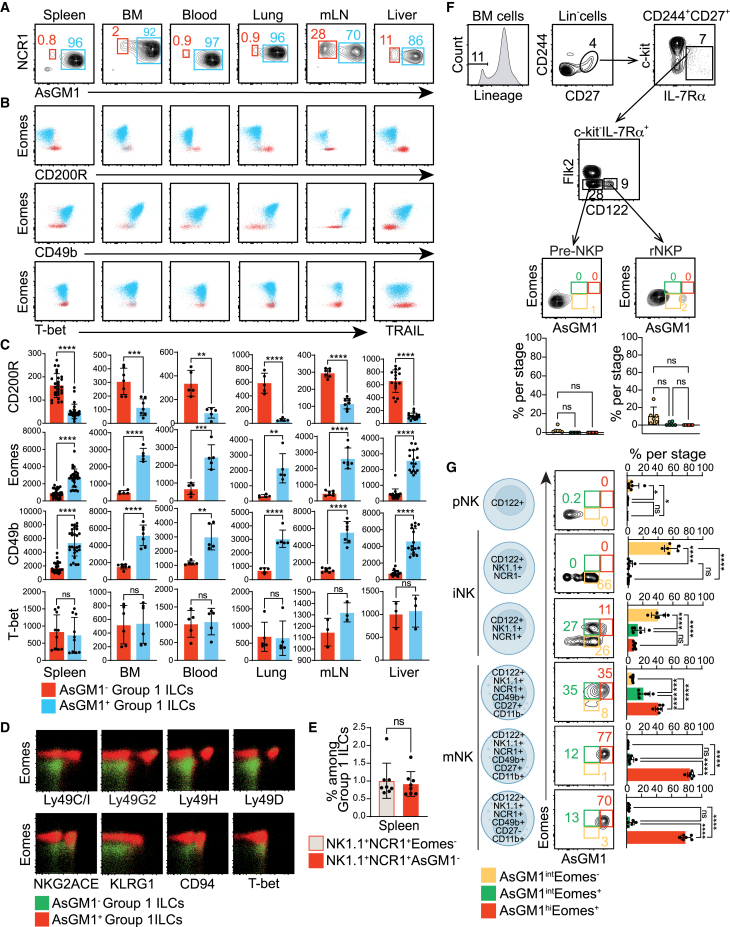
Mapping AsGM1 expression across Group 1 ILCs (A) Distribution of NCR1 vs. AsGM1 in CD3^−^NK1.1^+^ ILCs in the spleen, bone marrow (BM), blood, lung, mesenteric lymph nodes (mLN), and liver. (B) Profiles of Eomes, CD200R, CD49b, T-bet, and TRAIL in AsGM1^-^ and AsGM1^+^ ILCs across tissues. (C) Mean Fluorescence Intensity (MFI) for CD200R, Eomes, CD49b, and T-bet in AsGM1^-^ and AsGM1^+^ ILCs across tissues. (D) Comparison of activating and inhibitory NK cell markers in AsGM1^-^ and AsGM1^+^ ILCs in the spleen. (E) Proportion of Group 1 ILCs delineated as CD3^−^NK1.1^+^NCR1^+^Eomes^−^ vs. CD3^−^NK1.1^+^NCR1^+^AsGM1^-^ in the spleen. (F and G) Distribution of AsGM1 and Eomes expression across stages of NK cell development. (F) Gating strategy used to identify pre-NKP (Lin^−^CD244^+^CD27^+^c-kit^-^IL-7Rα^+^Flk2^−^CD122^-^) and rNKP (Lin^−^CD244^+^CD27^+^c-kit^-^IL-7Rα^+^Flk2^+^CD122^-^) cells.^32^ The lineage cocktail includes antibodies against CD19, Ly6D, CD3, NK1.1, and CD11b. (G) Stages within the immature (iNK) and mature (mNK) cell compartments are indicated by phenotypic markers listed. Distribution of AsGM1 versus Eomes expression within each developmental stage, with the proportions of AsGM1^int^Eomes^−^ (Yellow), AsGM1^int^Eomes^+^ (Green) and AsGM1^hi^Eomes^+^ (Red) subsets shown for each stage indicated in (F and G). Data represent 2–3 independent experiments: spleen (*n* = 27), BM (*n* = 6), blood (*n* = 5), lung (*n* = 5), mLN (*n* = 7), and liver (*n* = 16). Statistical analysis was performed using unpaired t-tests (C and E) and one-way ANOVA (F and G), with significance indicated by ∗*p* < 0.05, ∗∗*p* < 0.01, ∗∗∗*p* < 0.001, ∗∗∗∗*p* < 0.0001. Error bars represent mean +s.d. Refer to Figure S3 for additional information. Refer to Figure S3 for additional information.

Since AsGM1 has been linked to cytotoxic functions, we examined its expression across innate and adaptive lymphoid cells to determine whether its distribution aligns with cytotoxic potential within these immune cell populations (Figure 1A). AsGM1 was found primarily expressed in Group 1 ILCs, with little to no expression in other lymphocytes, suggesting a unique compartmentalization that may provide a basis for discriminating between ILC1s and NK cells. Given this distinctive expression pattern, we mapped the distribution of AsGM1 among Group 1 ILCs across a spectrum of anatomical sites—including lymphoid (bone marrow [BM], mesenteric lymph nodes [mLN] and spleen), circulatory (blood), non-lymphoid (liver), and mucosal (lung and small intestine lamina propria [siLP]). This spatial analysis revealed a clear demarcation of AsGM1 expression, effectively segregating Group 1 ILCs into two well-defined subsets: AsGM1^-^ and AsGM1^+^ (Figure 2A). AsGM1^+^ ILCs were predominantly found in the Group 1 ILC niches of the spleen, BM, blood, and lungs, while AsGM1^-^ ILCs were more abundant in the liver and mLN (Figure 1B; Figure S1), and distinctly predominant in the siLP (Figure 1B; Figures S2A and S2B). This distribution pattern was validated by anti-AsGM1 antibody treatment, which resulted in significant depletion of Group 1 ILCs in the spleen, liver, and bone marrow, whereas only a modest decrease was observed in the intestine, a site that harbors a substantial population of AsGM1^-^ Group1 ILCs (Figure 1C). These observations, further supported by the overlap between AsGM1^-^ ILCs and ILC1 frequencies (Figure 2E), underscore a parallel between the anatomical distribution of AsGM1-segregated ILCs and that of ILC1s and NK cells.^33^ Subsequent analysis contrasting the expression profiles of AsGM1-segregated ILCs with the known marker signatures of ILC1s and NK cells, also revealed a significant parallel (Figures 2B and 2C; Figure S2C). Precisely, AsGM1^-^ ILCs across examined sites displayed an ILC1 profile characterized by the expression of CD200R and T-bet (with liver ILCs additionally expressing TRAIL) while lacking Eomes and CD49b. Conversely, AsGM1^+^ ILCs exhibited traits of NK cells, marked by the expression of Eomes, T-bet, and CD49b and the lack of CD200R and TRAIL. AsGM1^+^ ILCs also displayed a wide array of activating and inhibitory markers including Ly49C/I, Ly49D, Ly49G2, Ly49H, NKG2ACE, KLRG1, and CD94. In contrast, AsGM1^-^ ILCs were devoid of this NK cell signature, conclusively aligning them with the characteristic profile of ILC1s (Figure 2D).

To determine whether AsGM1 signature in NK cells occurs at a mature stage or at a precursor level, we mapped its expression throughout NK cell maturation, including precursor (Pre-NK and rNKP),^32^ immature (iNK), and mature (mNK) stages (Figures 2F and 2G; Figure S3). Our results traced AsGM1 expression to early development, first detected at the immature NK (iNK) stage—a crucial phase characterized by the initial detection of Eomes^48^^,^^49^ and inhibitory and activating receptors,^50^^,^^51^ essential for functional maturation. Although Eomes and AsGM1 first appear at the iNK stage, our data indicates that AsGM1 expression precedes that of Eomes, occurring in NK1.1^+^NCR1^-^ iNK cells, while Eomes is detected in the subsequent NK1.1^+^NCR1^+^ iNK stage^52^^,^^53^ (Figure 2G; Figure S3). This sequential expression pattern at the iNK stage—which is known to foster immature NK cells as well as ILC1 precursors^54^^,^^55^—underscores a critical juncture where AsGM1^+^ cells become likely poised to diverge into the NK cell lineage, with the subsequent Eomes expression further driving NK cell lineage specification.^48^^,^^49^ Collectively, our data highlight the remarkable specificity of AsGM1 in delineating NK and ILC1 identities at a mature stage while suggesting its potential role in segregating their developmental trajectories at an immature stage.

### Transcriptomic profiling defines AsGM1-segregated innate lymphoid cells as bonafide natural killer cells and ILC1s

**Figure 3:**
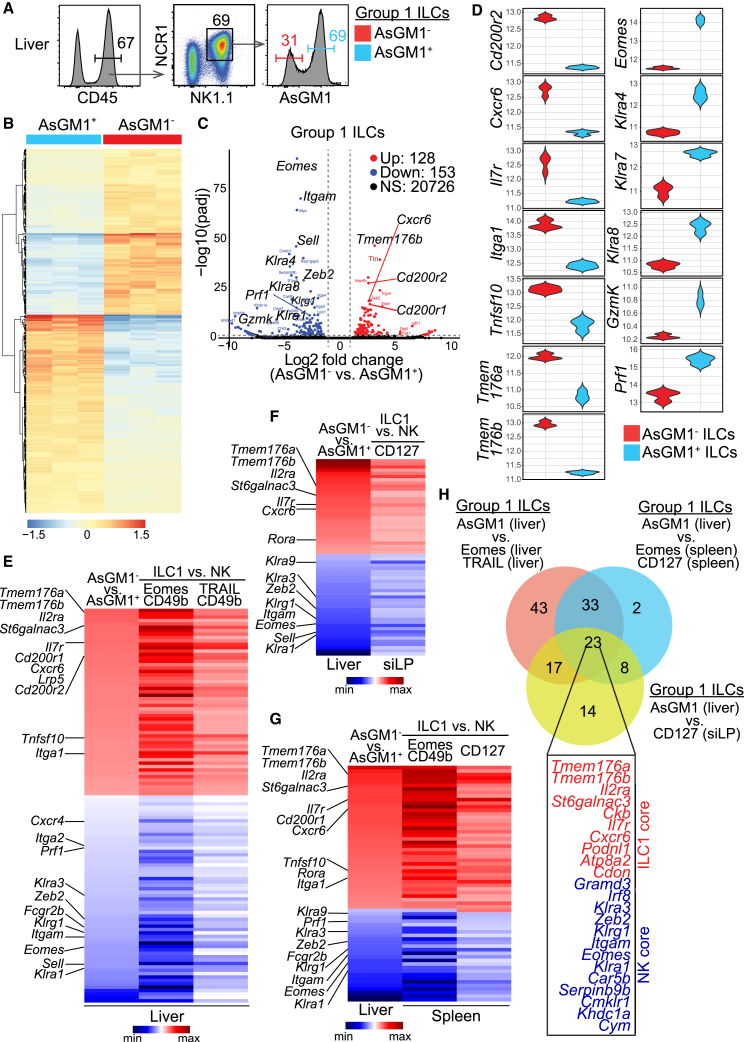
Profiles of AsGM1^-^ and AsGM1^+^ Group 1 ILCs match ILC1s and NK cells (A–D) Transcriptomic profiling of AsGM1-segregated Group 1 ILCs. (A) Gating strategy for sorting AsGM1^-^ and AsGM1^+^ Group 1 ILCs from the liver of *Rag2*^*−/−*^ mice. (B) Heatmap showing the differential gene expression between AsGM1^-^ and AsGM1^+^ Group 1 ILCs. (C) Volcano plot contrasting gene expression between AsGM1^-^ and AsGM1^+^ Group 1 ILCs, highlighting differentially expressed genes (DEGs) based on significance (*p*-values <0.05) and magnitude of change (fold-change >1.5): up-regulated (red), down-regulated (blue), and non-significant changes (black). (D) Violin plots illustrating the expression patterns of DEGs associated with ILC1 (left) or NK cell (right) profiles. (E–G) Comparative analysis of DEGs from AsGM1-segregated Group 1 ILCs [log_2_(AsGM1^-^/AsGM1^+^)] against DEGs from existing datasets of ILC1 and NK cell subsets [log_2_(ILC1/NK)], identified based on marker combinations of Eomes and CD49b, TRAIL and CD49b, or CD127 across the liver^36^^,^^59^^,^^60^ (E), intestine^59^^,^^60^ (F), and the spleen^36^^,^^59^^,^^60^ (G). (H) Venn diagram illustrating the overlap in gene expression across Group 1 ILC profiles, distinguished by AsGM1 in the liver versus ILC1s and NK cells the liver,^36^^,^^59^^,^^60^ spleen,^36^^,^^59^^,^^60^ and intestine.^59^^,^^60^ A list of 23 genes at the intersection of all comparison is provided. Data represent *n* = 3. Refer to Figure S4 for additional information.

To conclusively establish the identity of AsGM1-segregated ILCs as bona fide ILC1s and NK cells, we used RNA-sequencing to compare their transcriptomic profiles (Figures 3A–3D). Using *Rag2*^*−/−*^ mice, selected for their enriched ILC populations resulting from the absence of adaptive immune cells, enabled precise isolation of AsGM1^-^ and AsGM1^+^ subsets within Group 1 ILCs in the liver, where the frequency of both subsets is notably high^56^ (Figure 3A). Notably, we found that AsGM1 expression in Group 1 ILCs was comparable between Rag-deficient and wild-type mice (Figure S4), consistent with prior reports showing similar ILC1 populations in hepatic tissues across these mice.^57^ The comparison of transcriptomic profiles between AsGM1^-^ and AsGM1^+^ subsets revealed a signature of 281 differentially expressed genes (DEGs) (Figure 3B). Among these DEGs, genes traditionally associated with ILC1s,^58^ such as *Cd200r1*, *Cd200r2*, *Cxcr6*, *Il7r*, *Itga1*, *Tmem176a*, *Tmem176b*, and *Tnfsf10*, were predominantly expressed in AsGM1^-^ ILCs but notably lacking in AsGM1^+^ ILCs. Conversely, genes characteristic of NK cells,^58^ including *Eomes*, *Prf1*, *Irf8*, *Gzmk*, *Klra4*, *Klra7*, and *Klra8*, were abundantly expressed in AsGM1^+^ ILCs but markedly reduced in the AsGM1^-^ subset (Figures 3C and 3D). To validate these findings, we cross-referenced our data with transcriptomic datasets from independent studies that delineated ILC1s and NK cells in the liver, spleen, and intestine by distinct expression patterns of Eomes, TRAIL, or CD127^36^^,^^59^^,^^60^ (Figures 3E–3G). Across all heatmaps, our analysis revealed a uniform pattern of differential gene expression, firmly establishing the identity of AsGM1^-^ and AsGM1^+^ Group 1 ILCs as authentic ILC1 and NK cell subsets, respectively.

Contrasting AsGM1-defined gene expression in the liver with 5 profiles identified by Eomes or TRAIL in the liver,^36^^,^^59^^,^^60^ Eomes or CD127 in the spleen,^36^^,^^59^^,^^60^ and CD127 in the intestine,^59^^,^^60^ revealed a consensus set of 23 genes, comprising 10 ILC1 core genes and 13 NK core genes. (Figure 3H). Within the intersecting ILC1 core, *Il7r*^59^ and *Ckb*,^61^ known to be expressed in ILC progenitors (ILCPs),^62^ supports the unique origin of ILC1s from ILCPs, diverging from the NK cell lineage. *Podnl1*,^61^^,^^63^ integral to the ILC1 core for cell adhesion and cellular matrix interactions,^64^ aligns with the predominant localization of ILC1s as tissue-residents—as opposed to the circulatory nature of NK cells. *St6galnac3*, a gene that encodes a sialyltransferase responsible for attaching sialic acid to glycoproteins and glycolipids,^65^ contrasts sharply with the sialic acid-deficient AsGM1 glycoprotein.^66^ Accordingly, the ubiquitous expression of *St6galnac3*^61^ in ILC1s outlines a distinct sialylation landscape that diverges ILC1s from the cytotoxic profile of NK cells, which is linked to AsGM1.^43^^,^^44^^,^^45^^,^^46^^,^^47^ Within the intersecting NK core, *Gramd3* expression aligns with its association with optimal cytotoxicity mediated by DNAM.^67^^,^^68^ *Serpinb9b*, a gene encoding a serine protease inhibitor, plays a key role in immunoregulation by obstructing Granzyme B activity, ensuring that NK cells execute targeted cytotoxic actions without self-inflicted damage.^69^ Its identification in our intersecting NK core,^48^^,^^59^^,^^61^ aligns with its pivotal role in protecting the cytotoxic function of NK cells, absent in ILC1s. *Cmklr1*, a chemokine receptor associated with the migration of human and murine NK cells to sites of tumors,^70^^,^^71^^,^^72^ emerged as a gene signature of NK cells,^71^ analogous to the integral *Cxcr6* expression in ILC1s. Lastly, it is unsurprising that *Itgam,*^58^^,^^61^ *Khdc1a,*^61^ and *Cym*^61^ genes emerged in our intersecting NK core, given their association with T-bet and/or Eomes.^48^

**Figure 4:**
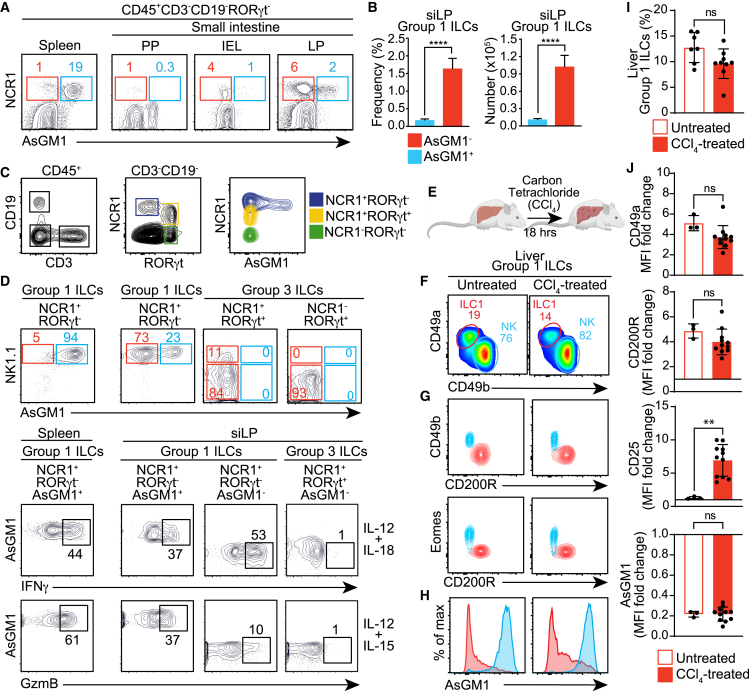
Stable AsGM1 signatures define Group 1 ILCs from different anatomical locations upon acute activation Patterns of AsGM1 expression in Group 1 ILCs from the intestine (A–D) and liver (E–J), subjected to *in vitro* and *in vivo* stimulations, respectively. (A) Pattern of AsGM1 expression within Group 1 ILCs in the spleen and small intestine Peyer’s patches (PP), intraepithelial lymphocytes (IEL), and lamina propria (siLP). (B) Frequency and numbers of AsGM1-segregated Group 1 ILCs within the siLP. (C) AsGM1 expression among intestinal Group 1 ILCs (NCR1^+^Rorγt^−^) and Group 3 ILCs (NCR1^+^Rorγt^+^ and NCR1^−^Rorγt^+^). (D) IFNγ and GzmB expression in AsGM1^-^ and AsGM1^+^ Group 1 ILCs in the siLP in response to stimulation with IL-12 plus IL-18 or IL-12 plus IL-15. Spleen NK cells and siLP ILC3s are used as controls. (E and F) Distribution of CD49a vs. CD49b within hepatic Group 1 ILCs before and 18 h after CCl_4_ treatment. (G) Overlay comparison of CD49b vs. CD200R and Eomes vs. CD200R distribution between ILC1s and NK cells before and after CCl_4_ treatment. (H) Patterns of AsGM1 expression in ILC1s and NK cells before and after CCl_4_ treatment. (I) Frequency of hepatic Group 1 ILCs before and after CCl_4_ treatment. (J) Mean Fluorescence Intensity (MFI) of CD49a, CD200R, CD25, and AsGM1 in ILC1s normalized to NK cells of untreated and CCl_4_-treated mice. Data represent 3 (A–D) and 2 (E–J) independent experiments, with *n* = 3–6 (A–D) and *n* = 3–11 (E–J). Statistical analysis was performed using unpaired t-test (B, I, and J), with significance indicated by ∗∗*p* < 0.01, ∗∗∗∗*p* < 0.0001. Error bars represent mean +s.d. Refer to Figure S5 for additional information.

To conclusively establish functional parallels—between AsGM1^-^ ILCs and ILC1s on one hand, and AsGM1^+^ ILCs and NK cells on the other—we examined the cytokine responses of both AsGM1^-^ and AsGM1^+^ Group 1 ILCs to IL-12 and IL-18, which induce IFNγ production,^73^ and IL-12 plus IL-15, which triggers Granzyme B (GzmB) expression.^74^ To this end, we used the siLP—a mucosal site enriched with NK cells, ILC1s, and ILC3s— which provides an optimal context for comparing AsGM1^-^ and AsGM1^+^ Group 1 ILCs, while also allowing ILC3s to serve as an internal control (Figures 4A–4D). While both AsGM1^-^ and AsGM1^+^ Group 1 ILCs exhibited comparable IFNγ output, underscoring functional similarities, the cytotoxic capability—evidenced by GzmB expression and the efficacy to kill YAC-1 cells—was specific to the AsGM1^+^ subset (Figure 4D; Figure S5). This distinction emphasizes the functional divergence within AsGM1-segregated ILCs, mirroring the functional dichotomy between ILC1s and NK cell subsets.^36^^,^^39^ These findings, stemming from integrated transcriptomic profiling and functional assays, introduce a new framework that adeptly distinguishes NK cells from ILC1s, via a single-marker approach predicated on cytotoxic potential.

### Stable AsGM1 expression in the continuum from homeostasis to acute response

The task of accurately distinguishing ILC1s from NK cells becomes increasingly challenging under physiological stress, where dynamic shifts in marker expression often occur.^18^^,^^20^^,^^29^^,^^34^^,^^35^^,^^36^^,^^75^ To evaluate the effectiveness of AsGM1 in resolving the heterogeneity of Group 1 ILCs under physiological stress or injury, we used a carbon tetrachloride (CCl_4_)-induced model of acute liver injury, known to trigger the acute activation of ILC1s in the liver.^76^ Following established protocols,^76^ C57BL/6 mice received an intraperitoneal injection of CCl_4_; after 18 h, hepatic Group 1 ILCs were analyzed to compare AsGM1 expression in steady state versus acute activation (Figures 4E–4J). We applied established markers to differentiate ILC1s (CD3^−^NK1.1^+^CD49a^+^CD49b^−^CD200R^+^) from NK cells (CD3^−^NK1.1^+^CD49a^−^CD49b^+^CD200R^−^) (Figures 4F and 4G). Additionally, we used CD25 as an indicator of activation to verify the immediate response of liver ILC1s to CCl4 treatment^76^ (Figure 4J). Using this experimental setup, we proceeded to assess the patterns of AsGM1 expression in response to acute activation. Our data showed that AsGM1 expression within Group 1 ILCs remained a reliable marker for distinguishing ILC1s from NK cells in CCl_4_-treated mice compared to control counterparts (Figures 4H and 4J). Specifically, ILC1s—whether in a resting state or under acute activation—maintained an AsGM1^-^ profile, while NK cells invariably showed an AsGM1^+^ profile. This remarkable stability, despite the physiological stress triggered by acute liver injury, underscored AsGM1’s unparalleled precision in delineating the binary classification of NK cells and ILC1s, bridging the continuum from steady state to immediate response with a clear demarcation.

### AsGM1 as a potential tool for tracing intra-lineage dynamics within group 1 innate lymphoid cells

**Figure 5:**
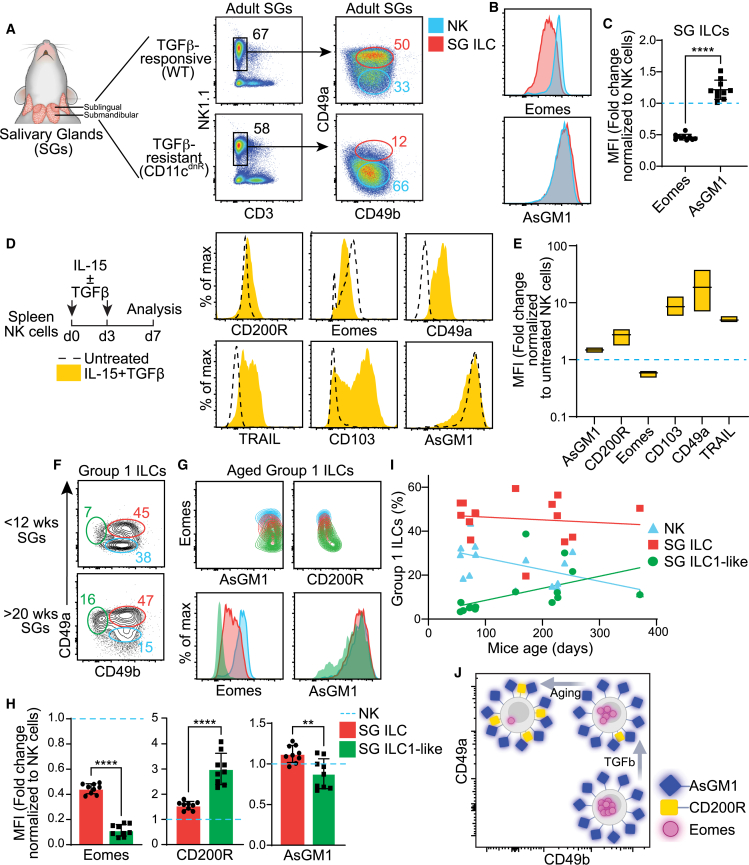
Mapping NK-to-ILC1 continuum in the salivary glands via AsGM1 (A) Distribution of CD49a vs. CD49b among Group 1 ILCs (CD45^+^CD3^−^NK1.1^+^) in the salivary glands (SGs) of adult wild-type (WT) and CD11c^dnR^ (TGFβR-resistant NK cells) mice. (B) Expression of Eomes and AsGM1 in CD49a^−^CD49b^+^ (NK cells) and CD49a^+^CD49b^+^ (SG ILCs) subsets from adult wild-type SGs. (C) MFI of Eomes and AsGM1 expression in SG ILCs relative normalized to NK cells in wild-type SGs. (D) Expression of CD200R, Eomes, CD49a, TRAIL, CD103, and AsGM1 in splenic NK cells before (dotted line) and after IL-15 plus TGFβ treatment (yellow). (E) MFI of AsGM1, CD200R, Eomes, CD103, CD49a, and TRAIL under IL-15 plus TGFβ treatment normalized to untreated NK cell controls. (F) Distribution of CD49a vs. CD49b in SG Group 1 ILCs in adult (2–4 months) and aged (5–12 months) wild-type mice. Gates indicate NK cells (blue), SG ILCs (red), and SG-ILC1-like cells (green). (G) Overlay of NK cells, SG ILCs and SG ILC1-like cells from aged SGs within the distribution of Eomes vs. AsGM1 and Eomes vs. CD200R. Expression of Eomes and AsGM1 is shown in overlayed cell subsets. (H) MFI of Eomes, CD200R, and AsGM1 in SG ILCs and SG ILC1-like cells normalized to NK cells in aged (5–12 months) mice. (I) Age-related frequency shifts in NK cells, SG ILCs, and SG ILC1-like cell subsets. (J) Schematic representation illustrating the stable expression of AsGM1 against the fluctuating expression of CD200R and Eomes in SG Group 1 ILCs, highlighting an age-associated continuum. Data represent 3 independent experiments: *n* = 11 (A–C), *n* = 3 (D and E), *n* = 13 (F–I) for wild-type mice, with *n* = 5 for CD11c^dnR^ mice (A). Statistical analysis was performed using unpaired t-test (C, H) and one-way ANOVA (E), with significance indicated by ∗∗*p* < 0.01, ∗∗∗∗*p* < 0.0001. Error bars represent mean +s.d.

An inherent challenge posed by cellular plasticity lies in the transcriptomic reprogramming it entails, which modifies cellular identities making it difficult to ascertain which cells are stable and which are plastic.^77^ Here, we aim to test whether AsGM1 can serve as a stable marker to trace NK cell derivatives. This hypothesis rests on the premise that AsGM1, a cellular membrane lipid component,^40^^,^^41^ remains unaffected by the transcriptomic reprogramming associated with NK-to-ILC1 transitions, allowing the reliable tracking of transitional states. To this end, we used a well-established *in vivo* model of TGFβ-driven NK-to-ILC1 plasticity in the salivary glands (SG),^18^ faithfully reproducing the documented conditions to determine whether transitional cells retain AsGM1 expression. Consistent with prior findings,^18^ our data confirmed the progression from a primarily NK cell phenotype (CD49a^−^CD49b^+^) in the SGs of CD11c^dnR^ mice^78^^,^^79^ (characterized by TGFβ-resistant NK cells) to a dominant ILC1-like profile (CD49a^+^CD49b^+^, known as SG ILCs) in wild-type SGs (TGFβ-responsive NK cells) (Figure 5A). During this transition, Eomes expression was downregulated, as evidenced by intermediate levels in SG ILCs. In contrast, AsGM1 expression remained stable, underscoring its potential as a reliable marker for tracking NK-to-ILC1 plasticity (Figures 5B and 5C). To further validate the stability of AsGM1, we used a well-established *in vitro* model of NK-to-ILC1 plasticity, which involves treating splenic NK cells with TGFβ plus IL-15 to induce their conversion into ILC1-like entities^19^ (Figures 5D and 5E). After a seven-day exposure to this cytokine milieu, the resultant cells exhibited a downregulation of Eomes along with an upregulation of CD49a, CD103, and TRAIL, mirroring outcomes of NK cell conversion associated with TGFβ in prior studies.^18^^,^^19^ Notably, AsGM1 expression remained constant throughout this transition, underscoring its stability during NK-to-ILC1 plasticity (Figures 5D and 5E).

Given that aging introduces qualitative alterations in the microenvironment,^80^ we focused on how these shifts affect NK-to-ILC1 plasticity within the aging SGs, postulating that the transformed milieu of an aged setting might lead to distinct plasticity outcomes. Within this unique context, AsGM1 emerged as a reliable marker, identifying a previously undescribed age-dependent ILC1-like state (AsGM1^+^CD200R^+^Eomes^−^CD49a^+^CD49b^−^), designated as SG ILC1-like cells (Figures 5F–5I). This finding extends the known spectrum of SG Group 1 ILC diversity to incorporate NK cells, SG ILCs, and the age-dependent SG ILC1-like cells, unified by their shared AsGM1 expression (Figures 5F–5H). The distinct gradients established by Eomes and CD200R expression among these three subsets, highlight an age-associated continuum of plasticity within the Group 1 ILC lineage, evolving from NK cells to SG ILCs, and ultimately to SG ILC1-like cells (Figure 5G). This progression, characterized by the reciprocal modulation of Eomes and CD200R together with stable AsGM1 expression, places SG ILC1-like cells at an advanced stage in the NK-to-ILC1 continuum, influenced by the unique aging microenvironment (Figure 5G). Additionally, age-related shifts in cell prevalence, highlighted by a decrease in NK cells and an increase in SG ILC1-like cells (Figure 5I), illustrate an age-dependent adaptation and reconfiguration of the SG Group 1 ILC landscape, distinctly captured by AsGM1 tracing (Figure 5J). This continuum depicted in aged SGs strikingly mirrors the transition from NK cells (CD49b^−^CD49a^−^) to intermediate ILC1s (CD49b^+^CD49a^+^) and to ILC1s (CD49b^−^CD49a^+^) in the tumor microenvironment,^19^ hinting at a unified adaptation strategy of Group 1 ILCs in aging and cancer, two different scenarios united by gradual timelines of cellular and molecular damage accumulation.

### Expanded landscape of group 1 innate lymphoid cells revealed by AsGM1 mapping during *T. gondii* infection

**Figure 6:**
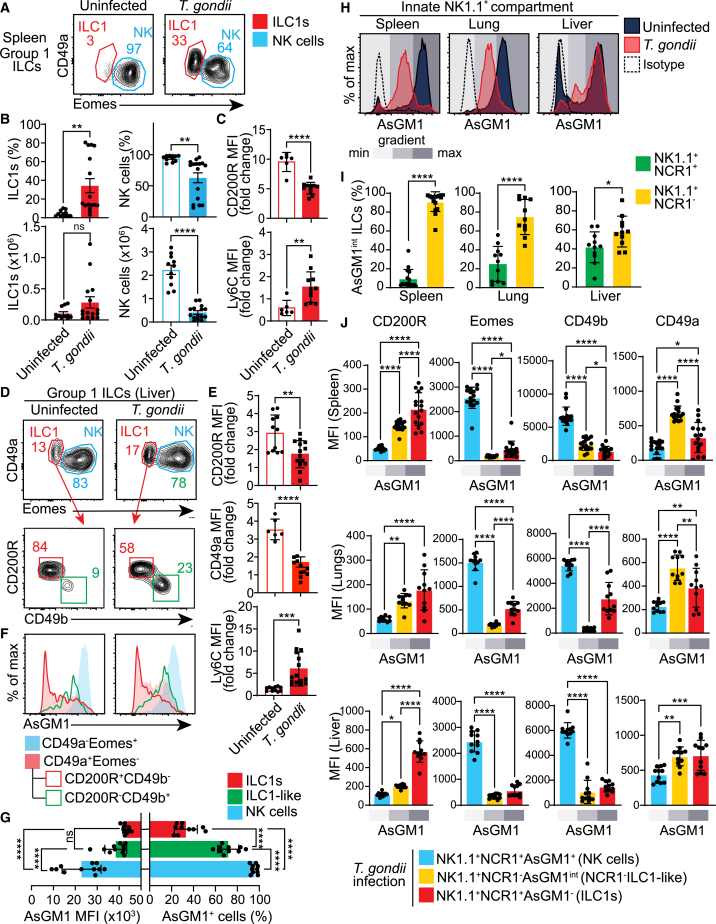
AsGM1 gradient driven by *T. gondii* infection captures two distinct ILC1-like subsets (A–J) Analysis of uninfected and *T. gondii*-infected mice 14 days post-infection. (A) Distribution of CD49a vs. Eomes among splenic Group 1 ILCs (CD45^+^CD3^−^NK1.1^+^NCR1^+^) from uninfected and *T. gondii*-infected mice. (B) Frequency and numbers of ILC1s and NK cells among splenic Group 1 ILCs from uninfected and *T. gondii*-infected mice. (C) MFI of CD200R and Ly6C in splenic ILC1s normalized to splenic NK cells from uninfected and *T. gondii*-infected mice. (D) Distribution of CD49a vs. Eomes among liver Group 1 ILCs from uninfected and *T. gondii*-infected mice. Distribution of CD200R vs. CD49b among liver ILC1s from uninfected and *T. gondii*-infected mice. (E) MFI of CD200R, CD49a, and Ly6C in liver ILC1s normalized to liver NK cells of uninfected and *T. gondii*-infected mice. (F) AsGM1 expression across liver Group 1 ILC subsets from uninfected and *T. gondii*-infected mice. (G) Frequency and MFI of AsGM1 in ILC1s, ILC1-like cells, and NK cells in the liver of *T. gondii*-infected mice. (H) AsGM1 expression in CD45^+^CD3^−^NK1.1^+^ ILCs from the spleen, lung, and liver of uninfected and *T. gondii*-infected mice. A graded gray bar represents the AsGM1 expression gradient, with dashed histograms indicating isotype control levels. (I) Distribution of NCR1^+^ and NCR1^-^ ILC subsets among NK1.1^+^AsGM1^int^ ILCs across spleen, lung, and liver in *T. gondii*-infected mice. (J) MFI of CD200R, Eomes, CD49b, and CD49a in NK1.1^+^NCR1^−^AsGM1^int^ ILCs compared with NK cells and ILC1s in the spleen, lung, and liver of *T. gondii*-infected mice. A graded gray bar indicates AsGM1 expression. Data represent 2 independent experiments, with *n* = 6–11 uninfected mice and *n* = 11–16 infected mice. Statistical analysis was performed using unpaired t-test (B, C, E, and I) and one-way ANOVA (G and J), with significance indicated by ∗*p* < 0.05, ∗∗*p* < 0.01, ∗∗∗*p* < 0.001, ∗∗∗∗*p* < 0.0001. Error bars represent mean ± s.e.m. (B) and mean ± s.d. (C, E, G, I, and J). Refer to Figures S6–S8 for additional information.

To assess the broader application of AsGM1 tracing across different immunological contexts, we employed *T. gondii* infection—a well-documented model of NK-to-ILC1 plasticity—to evaluate its effectiveness in capturing infection-driven plasticity.^22^ Following established protocols, we inoculated C57BL/6 mice with *T. gondii* and assessed alterations within the Group 1 ILC lineage in the spleen, lung, and liver 14 days post-infection^22^ (Figure 6). In line with prior findings in this model, we observed an expansion of the ILC1 pool and a concurrent reduction of the NK cell pool post-infection, consistent with previously described shifts^22^ (Figures 6A and 6B). Additional analysis demonstrating reduced levels of CD200R and CD49a, alongside the upregulation of Ly6C on ILC1s—key indicators of plasticity^22^—further substantiates this phenotypic shift (Figures 6C–6E; Figure S6). Further scrutiny of the liver ILC1 pool (NK1.1^+^NCR1^+^CD49a^+^Eomes^−^) from *T. gondii*-infected mice revealed a peculiar subset with NK cell characteristics, including the lack of CD200R and the expression of CD49b (Figure 6D). This subset displayed an atypical ILC1 profile characterized by NK1.1^+^NCR1^+^CD49a^+^Eomes^−^CD200R^−^CD49b^+^ phenotype, along with intermediate AsGM1 expression (AsGM1^int^), indicative of a transitional state potentially originating from NK cells (Figure 6F; Figures S7A–S7C). Mapping AsGM1 expression across Group 1 ILCs has thus unveiled a distinct gradient, capturing three subsets 14 days post-infection: conventional NK cells (AsGM1^+^), classical ILC1s (AsGM1^-^), and, notably, transitional ILC1-like cells,^22^ which we identified by their intermediate AsGM1 expression (AsGM1^int^) (Figure 6G).

An additional subset of AsGM1^int^ ILC1-like cells, distinguished by the lack of NCR1, was identified within the Group 1 ILC population across all sampled tissues (Figure S7D), with a notable predominance in the infected spleens and lungs (Figures 6H and 6I; Figure S8A). This distinct subset displayed a peculiar phenotype, characterized by NK1.1 and CD49a expression, lack of NCR1, CD49b, and Eomes, and intermediate expression of CD200R and AsGM1, consistent with a transitional stage in the NK-to-ILC1 continuum driven by *T. gondii* infection (Figure 6J). This is further substantiated by infection-associated shifts in cell prevalence, marked by a reduction in NK cells and a rise in AsGM1^int^NCR1^-^ ILC1-like cells (Figures S8B and S8C), demonstrating a significant, infection-driven reconfiguration of the Group 1 ILC landscape. This newly characterized population, which is likely overlooked due to the lack of NCR1, introduces a fourth subset to the Group 1 ILC spectrum, expanding its diversity to include two homeostatic (AsGM1^-^ ILC1s and AsGM1^+^ NK cells) subsets and two infection-driven ILC1-like states (AsGM1^int^NCR1^+^ and AsGM1^int^NCR1^-^), all distinctly captured by the AsGM1 expression gradient.

## Discussion

The evolution from a monolithic view of NK cells to a diverse framework—Group 1 ILCs— marked a pivotal shift in our understanding of the innate lymphoid system’s architecture.^81^ Initially, ILC1s were conflated with NK cells due to shared core markers—NK1.1, NCR1, T-bet, and IFNγ—that blurred their unique identities.^24^ The recognition of Group 1 ILCs as a diverse lineage, including both cytotoxic NK cells and non-cytotoxic ILC1s, necessitated a prompt reevaluation of their unique contributions to immunity and pathology. However, the inherent similarity^24^^,^^81^ and plasticity between NK cells and ILC1s^18^^,^^19^^,^^20^^,^^21^^,^^22^^,^^23^ obstructed their precise delineation and the elucidation of their unique contributions to immune regulation. This study introduces an innovative paradigm, exploiting the unique association of AsGM1 with cytotoxic attributes^43^^,^^44^^,^^45^^,^^46^^,^^47^—absent in ILC1s^33^^,^^36^—as a definitive criterion to distinguish ILC1s from NK cells. By prioritizing cytotoxic potential as the cardinal differentiator, our AsGM1-centric approach achieved accurate delineation, unveiling a precise view of the Group 1 ILC landscape and its remarkable plasticity across the health-disease continuum.

An ideal demarcation marker is (i) surface bound to preserve live cell integrity and functional potential, (ii) exhibits consistency across tissues and physiological contexts, and (iii) facilitates precise subset separation to minimize cell subset contamination risk. Against these benchmarks, AsGM1 emerged as a superior demarcating marker for Group 1 ILCs, surpassing traditional markers in precision and reliability. Its ability to distinguish ILC1s from NK cells across a spectrum of tissues -lymphoid, non-lymphoid, and mucosal was validated, with functional assays demonstrating its accuracy in identifying cytotoxic versus non-cytotoxic cellular identities within the Group 1 ILC lineage. RNA-seq analysis of AsGM1^-^ and AsGM1^+^ Group 1 ILCs confirmed their distinct identities as authentic ILC1s and NK cells, respectively. Corroborated by existing transcriptomic datasets, our findings established the efficacy of AsGM1 in segregating ILC1 and NK cell populations with equivalent accuracy to traditional multi-marker strategies.^36^^,^^59^ Likewise, extended analysis incorporating existing multi-tissue datasets^36^^,^^59^ revealed a uniform core transcriptomic signature associated with AsGM1, underscoring its potential as a versatile, site agnostic marker for the systematic delineation of NK cells and ILC1s. This capability extends beyond steady state classifications, adeptly capturing the binary classification of NK cells and ILC1s during immediate immune responses, as demonstrated by our findings in the acute liver injury model. However, the landscape of cellular identity within the Group 1 ILC pool becomes markedly more complex in chronic infection scenarios, such as those driven by *Toxoplasma gondii*,^22^ or within specific homeostatic contexts such as the salivary glands.^18^ In these cases, the binary classification of NK cells and ILC1s is disrupted by intra-lineage plasticity, namely NK-to-ILC1 plasticity, leading to the generation of transitional states that blur the boundaries between NK cells and ILC1s, resulting in a continuum of identities.^18^^,^^22^ Remarkably, AsGM1 expression across these conditions consistently marked ILC1-like entities, previously demonstrated to arise from NK cell transitions driven by factors such as TGFβ^19^ or T. gondii infection,^23^ effectively addressing the current challenges in distinguishing stable from transitional states.

NK-to-ILC1 plasticity is increasingly recognized as an important mechanism of adaptation characterized by the cellular reprogramming of NK cells into entities sharing ILC1 attributes.^18^^,^^19^^,^^20^^,^^21^^,^^22^^,^^23^^,^^24^ It involves the downregulation of NK cell markers (Eomes or CD49b) and upregulation of ILC1 markers (CD200R, CD49a, CD103, or TRAIL), resulting in a significant reduction of cytotoxic capabilities. These reprogrammed NK cells, now closely resembling ILC1s, shift their functional priority toward maintaining tissue integrity over executing direct cytotoxic actions, underscoring the formidable challenge of discerning the true identity of ILC1-like entities—whether they are authentic ILC1s or the products of NK cell plasticity. Validated in both TGFβ-driven^18^ and infection-driven^22^ models of NK-to-ILC1 plasticity, our findings suggest that AsGM1 may serve as a pivotal marker for identifying these cellular transitions, reflecting a shift in cellular identity and function. Building on TGFβ-driven NK-to-ILC1 transitions previously demonstrated in adult SGs,^18^ we identified these cellular states as AsGM1^+^ subsets, including NK cells (AsGM1^+^) in the absence of TGFβ and SG ILCs (AsGM1^+^) in its presence. Extending this framework to aging, we uncovered a distinct, SG ILC1-like subset (AsGM1^+^Eomes^−^CD200R^+^CD49b^−^CD49a^+^), suggesting additional age-associated dynamics within the Group 1 ILC compartment. This age-dependent subset closely mirrors classical ILC1 profile, nearly to the point of potential misidentification. However, an unorthodox expression of AsGM1 at this juncture rather points to a state within the NK-to-ILC1 continuum,^19^ likely influenced by the specific aging microenvironment, which is shaped by significant shifts in epigenetic landscapes^82^ and cytokine profiles.^83^

Leveraging insights from our investigation into the Group 1 ILC dynamics in aging SGs, scRNA-seq analysis of SG Group 1 ILCs compellingly supports and further extends our findings at a transcriptomic level.^58^ This analysis delineated five clusters, including NK cells, two ILC1 clusters, and two transitional states, aligning with TGFβ-driven plasticity in adult SGs.^58^ Notably, PCA did not segregate these clusters; rather, it unveiled a continuum of cellular states. Further, Pseudotime analysis traced these clusters from NK cells to ILC1s via transitional stages, marked by the sequential downregulation of NK cell core markers and upregulation of ILC1-related genes. This trajectory aligns with the continuum we have observed through AsGM1 tracing in aged SGs—from AsGM1^+^ NK cells to AsGM1^+^ SG ILCs and onto AsGM1^+^ SG ILC1-like cells. In parallel, our analysis of Group 1 ILCs during *T. gondii* infection^22^ identified a unique AsGM1 expression gradient, sharply distinguishing these infection-driven changes from homeostatic-driven alterations observed in the SGs. This gradient provided insights into two ILC1-like states through intermediate AsGM1 expression, uncovering a previously characterized ILC1-like subset primarily observed in infected livers,^22^ and revealing a distinct ILC1-like subset lacking NCR1, predominantly observed in infected spleens and lungs. The downregulation of NCR1 on NK cells, a phenotype prevalent in cancer,^84^^,^^85^^,^^86^^,^^87^^,^^88^^,^^89^^,^^90^^,^^91^ and its association with impaired cytotoxicity and increased IFNγ production,^92^^,^^93^ likely reflects a strategic adaptation to *T. gondii* infection, prioritizing tissue integrity and control of inflammation over cytotoxic actions. These four distinct ILC1-like entities captured by AsGM1 expression in contexts of aging and infection illustrate the expanding universe of Group 1 ILCs which may strategically utilize NK-to-ILC1 plasticity to finely tune the equilibrium between pathogen defense and tissue homeostasis as needed. However, it should be noted that the exact nature of the unique NCR1^-^ cellular state—whether resulting from cellular plasticity or representing an exhausted state—remains to be determined.

The central point of this study lies in the relative resilience of AsGM1—a membrane lipid marker^40^^,^^41^^,^^42^— compared to the loss of Eomes in ILC1-like entities that were previously demonstrated to arise from NK cell plasticity driven by factors such as TGFβ^18^ or T. gondii infection.^22^ This persistent expression of AsGM1 in ILC1-like entities, despite potential functional reprogramming and associated shifts in protein expression, likely reflects the inherent characteristics of lipids, which are less affected by transcriptional changes that directly influence protein expression.^94^^,^^95^ The stability of lipids, crucial for membrane structure, is maintained through mechanisms such as enzymatic regulation and metabolic processes, rather than direct transcriptional control.^94^ Specifically, AsGM1 regulation through glycosyltransferases, rather than gene expression changes, underscores a distinct pathway of control.^95^ Consistent with its established link to cytotoxic functions,^43^^,^^44^^,^^45^^,^^46^^,^^47^ AsGM1 was traced to the immature stage of the NK cell developmental pathway, highlighting its early involvement at a crucial point of functional commitment.^48^^,^^96^ This early expression, together with its potential stability—arguably less affected by the transcriptional reprogramming that alters Eomes—suggests that AsGM1 could serve as a valuable marker for fate mapping NK-to-ILC1 transitions. The recognition of intra-lineage plasticity as a mechanism of adaptation across diverse contexts and species,^9^^,^^10^^,^^11^^,^^12^^,^^13^^,^^14^^,^^15^^,^^16^^,^^17^^,^^18^^,^^19^^,^^20^^,^^21^^,^^22^^,^^23^^,^^97^ leading to distinct transitional entities, underscores the potential value of our findings in refining the classification of entities within the NK-to-ILC1 spectrum. This framework suggests that AsGM1 may help identify transitional states in clinical settings, potentially informing therapeutic strategies aimed at modulating these cells’ functional properties.

The precise role of AsGM1 within cell membrane lipid rafts remains to be fully elucidated. AsGM1 may stabilize signaling complexes essential for granule polarization and degranulation, which are key to cell-mediated cytotoxicity.^98^ It may facilitate the polarization of cytolytic granules containing perforin and granzymes toward the immunological synapse, aiding directed exocytosis and efficient lysis of target cells.^99^ AsGM1 might also regulate calcium influx, critical for the mobilization and fusion of cytolytic granules with the plasma membrane,^100^ thereby enhancing cytotoxic molecule release. As our understanding of ILC plasticity progresses, the role of AsGM1 in this dynamic landscape merits further exploration. This is particularly relevant in scenarios where ILC1-like states have been observed to express granzyme C, suggesting potential cytotoxic functions.^101^ Whether AsGM1 is present in these cellular states, and whether its expression indicates sustained cytotoxic potential or merely reflects lineage heritage, remains to be determined.

### Limitations of the study

This study was conducted exclusively in a murine model due to constraints in acquiring human tissue samples. While this provides foundational insights, it is essential to validate whether AsGM1 can similarly segregate NK cells and ILC1s in human tissues. Future investigations are crucial to establish the clinical relevance of these findings and potentially enhance therapeutic strategies targeting these cell types in human immune disorders.

## Resource availability

### Lead contact

Further information and requests for resources and reagents should be directed to and will be made available upon reasonable request by the lead contact: Yasmina Laouar (ylaouar@umich.edu).

### Materials availability

This study did not generate new unique reagents.

### Data and code availability

• Data: Bulk RNA-sequencing data have been deposited at GEO as GSE269280 and are publicly available as of the date of publication.
• Code: This article does not report original code.
• Additional information: any additional information required to reanalyze the data reported in this article is available from the lead contact upon request.

## Acknowledgments

Library preparation and next-generation sequencing were carried out at the Advanced Genomics Core, University of Michigan. Analysis of RNA-seq data was performed by the Bioinformatics Core at the University of Michigan Medical School’s Biomedical Research Core Facilities (RRID:SCR_019168). We thank the staff and teams at these facilities for their assistance. Funding: The study was supported by 10.13039/100000060NIAID (R21 AI14010602 and R21AI138180 to Y.L. and X.S., and R01 AI120607 V.B.C.).

## Author contributions

H.C.F., A.L., and Y.L designed the experiments. H.C.F., X.S., F.O., D.C., Z.J., T.W., and T.S. conducted the experiments and analyzed the data. H.C.F., X.S., and F.O. performed the statistical analysis. H.C.F., A.L., and Y.L wrote the article and H.C.F., X.S., F.O., A.L., and Y.L. designed the figures. T.W., V.B.C., A.L., and Y.L. edited the article.

## Declaration of interests

The authors declare no competing interests.

## STAR★Methods

### Key resources table

**Table:** 

REAGENT or RESOURCE		SOURCE	IDENTIFIER
**Antibodies**			

Rat monoclonal anti-CD45		BioLegend	Cat#103139; RRID: AB_2562341
Rat monoclonal anti-CD3		BioLegend	Cat#100213; RRID: AB_493644
Mouse monoclonal anti-NK1.1		BioLegend	Cat#156510; RRID: AB_2876527
Mouse monoclonal anti-NK1.1		BioLegend	Cat#108730; RRID: AB_2291262
Rat monoclonal anti-NKp46		BioLegend	Cat#137630; RRID: AB_2616666
Rat monoclonal anti-NKp46		BioLegend	Cat#137603; RRID: AB_10552741
Rabbit polyclonal anti-Asialo-GM1		BioLegend	Cat#146004; RRID: AB_2563196
Rat monoclonal anti-CD200R		BioLegend	Cat#123910; RRID: AB_2244385
Rat monoclonal anti-CD49b		BioLegend	Cat#108922; RRID: AB_2561460
Armenian Hamster monoclonal anti-CD49a		BioLegend	Cat#142612; RRID: AB_2750219
Armenian Hamster monoclonal anti-CD103		BioLegend	Cat#121425; RRID: AB_2563690
Rat monoclonal anti-CD25		Invitrogen	Cat#25-0251-81; RRID: AB_469607
Rat monoclonal anti-Ly6C		BioLegend	Cat#128033; RRID: AB_2562351
Rat monoclonal anti-TRAIL		BioLegend	Cat#109314; RRID: AB_2721720
Mouse monoclonal anti-Ly49C/I		BD Biosciences	Cat#562055; RRID: AB_394750
Mouse monoclonal anti-Ly49H		BioLegend	Cat#144707; RRID: AB_2561743
Rat monoclonal anti-Ly49D		BD Biosciences	Cat#555313; RRID: AB_395724
Rat monoclonal anti-Ly49G2		Invitrogen	Cat#46-5781-82; RRID: AB_1834437
Rat monoclonal anti-NKG2ACE		BD Biosciences	Cat#550520; RRID: AB_393723
Syrian Hamster monoclonal anti-KLRG1		BioLegend	Cat#138417; RRID: AB_2563014
Rat monoclonal anti-CD94		Invitrogen	Cat#11-0941-82; RRID: AB_465161
Mouse monoclonal anti-T-bet		BioLegend	Cat#644806; RRID: AB_1595488
Rat monoclonal anti-Eomes		BioLegend	Cat#157706; RRID: AB_2888891
Rat monoclonal anti-RORγt		Invitrogen	Cat#12-6981-80; RRID: AB_10805392
Rat monoclonal anti-IFNγ		BioLegend	Cat#505809; RRID: AB_315403
Mouse recombinant anti-GzmB		BioLegend	Cat#372208; RRID: AB_2687032
Rat monoclonal anti-CD122		BD Biosciences	Cat#553361; RRID: AB_394808
Armenian Hamster monoclonal anti-CD27		Invitrogen	Cat#46-0271-80; RRID: AB_1834448
Rat monoclonal anti-CD11b		BioLegend	Cat#101207; RRID: AB_312790
Rat monoclonal anti-IFNγ		BioLegend	Cat#505809; RRID: AB_315403
Rat monoclonal anti-CD19		BioLegend	Cat#152403; RRID: AB_2629812
Rat monoclonal anti-CD11b		BioLegend	Cat#101205; RRID: AB_312788
Rat monoclonal anti-Ly-6D		BioLegend	Cat#138605; RRID: AB_11203901
Armenian Hamster monoclonal anti-CD3e		BD Biosciences	Cat#553062; RRID: AB_394595
Mouse monoclonal anti-NK1.1		BioLegend	Cat#156507; RRID: AB_2876526
Mouse monoclonal anti-CD244.2		BioLegend	Cat#133513; RRID: AB_2564340
Rat monoclonal anti-CD127 (IL-7Rα)		BioLegend	Cat#135031; RRID: AB_2564216
Rat monoclonal anti-CD117 (c-kit)		BioLegend	Cat#135111; RRID: AB_2131136
Rat monoclonal anti-CD112		BioLegend	Cat#123221; RRID: AB_2750454
Armenian Hamster monoclonal anti-CD135 (Flk2)		BioLegend	Cat#135313; RRID: AB_2562338
Armenian Hamster monoclonal anti-CD27		BioLegend	Cat#124239; RRID: AB_2810382
Armenian Hamster monoclonal anti-CD3ε		BioLegend	Cat#100303; RRID: AB_312668
Rabbit polyclonal anti-Asialo-GM1		FUJIFILM Wako	Cat#986–10001; RRID: AB_516844
Rabbit IgG Isotype Control		ThermoFisher	Cat#51-4616-82; RRID: AB_2848406
Fc receptor block (anti-CD16/CD32 clone 2.4G2)		This paper	N/A

**Bacterial and virus strains**			

*T. gondii* strain ME49		Kannan et al.^102^	N/A

**Chemicals, peptides, and recombinant proteins**			

Carbon tetrachloride (CCl_4_)		Sigma-Aldrich	Cat#02671
Peanut oil		Sigma-Aldrich	Cat#P2144
Collagenase IV		Sigma-Aldrich	Cat#11088866001
DNase I		Sigma-Aldrich	Cat#10104159001
Percoll		Sigma-Aldrich	Cat#17089101
IL-12		Peprotech	Cat#210-12
IL-18		R&D systems	Cat#B002-5
IL-15		Peprotech	Cat#210-15
Golgi Stop		BD Biosciences	Cat#512092KZ
TGF-β		R&D systems	Cat#240-B
LIVE/DEAD™ Fixable Aqua Dead Cell Stain Kit		Invitrogen	Cat#L34965A
ViaFluor™ CFSE Cell Proliferation Kit		Biotium	Cat#30050

**Critical commercial assays**			

RNeasy Plus Mini Kit		QIAgen	Cat#74134
DNA-free kit		Ambion	Cat#AM1906
BioAnalyzer		Agilent	Cat#5067-1513
SMART-Seq Stranded kit		Takara	Cat#634444
Qubit hsDNA		Thermofisher	Cat#Q33231
LabChip		Perkin Elmer	Cat#CLS1444006
EasySep™ Mouse Streptavidin RapidSpheres™ Isolation Kit		Stemcell	Cat#19860

**Deposited data**			

Bulk RNA-sequencing data		This paper	GEO: GSE269280
Spleen and liver Group 1 ILCs		Weizman et al.^36^	GEO: GSE103901
Spleen, liver, and intestinal Group 1 ILCs		Robinette et al.^59^^,^^60^	GEO: GSE37448

**Experimental models: Cell lines**			

Mouse: YAC-1 cells		ATCC	ATCC: TIB-160

**Experimental models: Organisms/strains**			

Mouse: C57BL/6J		The Jackson Laboratory	Strain #:000664; RRID: IMSR_JAX:000664
Mouse: B6.Cg-Rag2tm1.1Cgn/J		The Jackson Laboratory	Strain #:008449; RRID: IMSR_JAX:008449
Mouse: B6.Cg-Tg(Itgax-TGFBR2)1Flv/J		Laouar et al.^78^	Strain #: 008378; RRID: IMSR_JAX 008378

**Software and algorithms**			

Graphpad Prism 10		Graphpad Software	RRID: SCR_002798
FlowJo 10		FlowJo LLC	RRID: SCR_008520
Cutadapt v2.3		Martin^103^	https://doi.org/10.14806/ej.17.1.200
FastQC v0.11.8		Simons	https://www.bioinformatics.babraham.ac.uk/projects/fastqc/
Fastq Screen v		Wingett et al.^104^	https://f1000research.com/articles/7-1338
STAR v2.7.8a		Dobin et al.^105^	https://doi.org/10.1093/bioinformatics/bts635
RSEM v1.3.3		Li et al.^106^	https://doi.org/10.1186/1471-2105-12-323
Multiqc v1.7		Ewels et al.^107^	https://doi.org/10.1093/bioinformatics/btw354
DESeq2		Love et al.^109^	https://doi.org/10.1186/s13059-014-0550-8
dplyr		Wickham	https://dplyr.tidyverse.org/
pheatmap		Kolde	https://cran.r-project.org/web/packages/pheatmap/pheatmap.pdf
ggplot2		Wickham	https://link.springer.com/book/10.1007/978-3-319-24277-4
ggrepel		Slowikowski	https://cran.r-project.org/web/packages/ggrepel/ggrepel.pdf
ggbreak		Xu et al.^108^	https://doi.org/10.3389/fgene.2021.774846
R version 4.3.0		The R Foundation	https://www.r-project.org/
bioinfokit		Bedre	https://github.com/reneshbedre/bioinfokit
VennDiagram		The R Project for Statistical computing	https://cran.r-project.org/web/packages/VennDiagram/index.html

### Experimental model and study participant details

#### Mice

B6 (C57BL/6) and *Rag2*^*−/−*^ (B6.Cg-Rag2tm1.1Cgn/J) mice were purchased from Jackson Laboratories and maintained at the University of Michigan. CD11c^dnR^ (B6.Cg-Tg(Itgax-TGFBR2)1Flv/J) transgenic mice^78^^,^^79^ were maintained at the University of Michigan. Adult male and female mice aged between 6 and 52 weeks, were used in this study. All mice were maintained at the University of Michigan in specific pathogen-free housing at 22°C. Littermates of the same sex were randomly assigned to experimental groups. The University of Michigan approved the use of rodents through the criteria in the Guide for the Care and Use of Laboratory Animals from the National Institutes of Health. All procedures described in this study were approved by IACUC under the ethical animal protocol # 00012457.

### Method details

#### Carbon tetrachloride (CCl_4_) acute liver injury

Adult C57BL/6 mice were injected intraperitoneally with 10μL of 10% CCl_4_ (02671; Sigma) diluted in peanut oil (P2144; Sigma) per gram of body weight.^76^ Eighteen hours post-treatment, livers were perfused with cold PBS and processed for analysis.

#### Parasitic infection

*T. gondii* ME49 strain tachyzoites were cultured in primary human foreskin fibroblasts (HFF) in Dulbecco’s modified Eagle’s medium (DMEM) supplemented with 10% Cosmic Calf serum (Hyclone), 20 mM HEPES, 5 μg/mL penicillin/streptomycin and 2 mM L-glutamine. Infected monolayers were scraped, ruptured to release the parasites by passage through a syringe with a 22g needle, and tachyzoites were recovered after passage through a 3 μm filter in PBS and one wash in PBS. Adult female C57BL/6 mice were injected intraperitoneally with 200 ME49 strain tachyzoites in PBS.^22^ Mice were euthanized 14–15 days post infection and perfused with cold PBS. Spleens, lungs, and livers were processed for analysis.

#### *In vivo* Anti-AsGM1 treatment

Rabbit Anti-AsGM1 antibody (FUJIFILM Wako) was diluted in 1 mL of sterile water, and 50 μL was administered intraperitoneally to adult C57BL/6 mice on days 0 and 2. Control mice received two doses of PBS on the same schedule.

#### Organ processing

Livers were harvested after perfusion with cold PBS and digested in 5% FBS medium (RPMI) supplemented with 0.01% collagenase IV (Sigma) and 0.001% DNase I (Sigma) for 30 min at 37°C under agitation (250 rpm). Liver mononuclear cells were obtained using Percoll gradient (Thermo Fisher) at the interface between 33% and 70% layers. Lungs were digested in 15 mL total of 10% FBS medium (RPMI) supplemented with 1 mg/mL collagenase IV and 0.5 mg/mL DNase I for 30 min at 37°C under agitation (250 rpm). Salivary (sublingual and submandibular) glands were digested in 10mL total of 5% FBS medium (RPMI) supplemented with 1 ul HEPES, 5mM CaCl_2_, 0.5 mg/mL collagenase IV and 0.1 mg/mL DNase I for 60 min at 37°C under agitation (250 rpm). Salivary gland mononuclear cells were obtained using Percoll gradient at the interface between 40% and 70% layers.^18^ To isolate intestinal cells, small intestine segments were first removed, and Peyer’s patches excised. After discarding and washing gut content, the small intestines were cut into small pieces and incubated in HBSS supplemented with 5% FBS, 2 mM EDTA (Fisher Scientific), 1 mM DTT (American Bioanalytical), and 10 μg/mL Gentamicin (Gibco) for 20 min at 37°C under agitation (250 rpm) to dissociate epithelial cells. Intraepithelial lymphocytes (IEL) were isolated from the supernatants by filtering through nylon wool (Polysciences) and discontinuous Percoll (GE Life Sciences) gradient at the interface between 33% and 70% layers. After a step of washing, intestinal pieces were digested in 5% FBS medium (RPMI) supplemented with 1 mg/mL collagenase D (Roche) and 0.5 mg/mL DNase I (Sigma) for 60 min at 37°C under agitation (250 rpm). Lamina propria mononuclear cells were obtained by Percoll gradient at the interface between 33% and 70% layers. To harvest PBMCs, blood was drawn by cardiac puncture after euthanasia and placed in a tube with EDTA to prevent clotting. Mononuclear cells obtained from livers, lungs, salivary glands, small intestine lamina propria, and IEL, as well as cells harvested from spleens, bone marrow, mesenteric lymph nodes, Peyer’s patches, and blood were filtered through 100μm cell strainer at final step before used for analysis. Red cells in the bone marrow, spleen, and blood were lysed using red cell lysis buffer (Invitrogen).

#### *In vitro* stimulation

Cells from spleens and small intestine lamina propria were stimulated with 5 ng/mL IL-12 (Peprotech) plus 25 ng/mL IL-18 (R&D Systems) or 5 ng/mL IL-12 (Peprotech) plus 50 ng/mL IL-15 (Peprotech) for 4–16 h at 37C with the protein-transporter inhibitor Golgi Stop (BD Biosciences) added during the last 4 h of culture. Stimulated cells were analyzed for intracellular expression of IFNγ and GzmB. When indicated, cells from C57BL/6 and CD11c^dnR^ spleens were cultured for 7 days with 10 ng/mL IL-15 (Peprotech) supplemented with or without 10 ng/mL TGFβ1 (R&D). Cytokines were added on days 0 and 3, and cells were analyzed on day 7 using LIVE/DEAD Fixable Aqua Dead Cell Stain Kit (Invitrogen) to exclude dead cells.

#### Flow cytometry and cell sorting

Single-cell suspensions from the liver, lung, salivary glands, small intestine lamina propria, PPs, IEL, spleen, bone marrow, blood, and mesenteric lymph nodes were first treated with Fc receptor block (anti-CD16/CD32 clone 2.4G2) produced by HB-197 hybridoma cell line from athymic nude mice in the Hybridoma core facility at the University of Michigan. The antibodies were then purified using NAb Protein A/G Spin kit (Thermofisher; Cat#89980). The cells were then stained using the following fluorochrome-conjugated antibodies purchased from Biolegend, eBioscience, or BD Biosciences: anti-CD45 (30-F11), anti-CD3 (17A2), anti-NK1.1 (S17016D and PK136), anti-NCR1 (29A1.4), anti-AsGM1 (Poly21460), anti-CD200R (OX-110), anti-CD49b (DX5), anti-CD49a (HMa1), anti-CD103 (2E7), anti-CD25 (7D4), anti-Ly6C (HK1.4), anti-TRAIL (N2B2), anti-Ly49C/I (5E6), anti-Ly49H (3D10), anti-Ly49D (4E5), anti-Ly49G2 (4D11), anti-NKG2ACE (20d5), anti-KLRG1 (2F1/KLRG1), anti-CD94 (18d3), anti-T-bet (4B10), anti-Eomes (W17001A), anti-RORγt (B2D), anti-IFNγ (XMG1.2), anti-GzmB (NGZB), anti-CD122 (TM-BETA 1), anti-CD27 (LG.7F9), and anti-CD11b (M1/70). For intracellular staining, cells were fixed and permeabilized using FOXP3/Transcription factor fixation/permeabilization kit (Invitrogen). Stained cells were acquired on BD FACSCanto or BD LSRFortessa flow cytometers and analysis was performed using FlowJo software. For cell sorting, liver mononuclear cells were stained with anti-CD45, anti-NK1.1, anti-NCR1, and anti-AsGM1 antibodies. Group 1 ILCs were sorted based on AsGM1 expression as follows: AsGM1^+^ ILCs; CD45^+^NK1.1^+^NCR1^+^AsGM1^+^ and AsGM1^-^ ILCs; CD45^+^NK1.1^+^NCR1^+^AsGM1^-^. Cells were sorted on FACSAria High-Speed Cell Sorter at the flow cytometry core facility (University of Michigan).

#### RNA preparation and sequencing

Total RNA was isolated from AsGM1^+^ and AsGM1^-^ ILC subsets using the RNeasy Plus Mini Kit (QIAgen) according to the manufacturer’s procedure. RNA samples were treated with the DNA-free kit (Ambion) to remove any traces of genomic DNA. RNA was assessed for quality using the BioAnalyzer (Agilent #5067-1513). The SMART-Seq Stranded kit (Takara #634444) was used for cDNA synthesis and amplification, ZapR-mediated ribosomal RNA depletion, and library preparation from 1.8 ng of total RNA. Final libraries were checked for quality and quantity by Qubit hsDNA (Thermofisher #Q33231) and LabChip (PerkinElmer #CLS1444006). The samples were pooled and sequenced on the Illumina NovaSeq S4 Paired-end 150bp, according to manufacturer’s protocols.

#### RNA-seq analysis

The reads were trimmed using Cutadapt v2.3.^103^ FastQC v0.11.8 was used to ensure the quality of data. Fastq Screen v was used to screen for various types of contamination.^104^ Reads were mapped to the reference genome GRCm38 (ENSEMBL), using STAR v2.7.8a^105^ and assigned count estimates to genes with RSEM v1.3.3.^106^ Alignment options followed ENCODE standards for RNA-seq. Multiqc v1.7 compiled the results from several of these tools and provided a detailed and comprehensive quality control report.^107^ Data were pre-filtered to remove genes with less than 10 counts in total from all samples. Differential gene expression analysis was performed using DESeq2^109^ using a negative binomial generalized linear model (thresholds: log2 fold change >1 or < -1, Benjamini-Hochberg FDR (Padj) < 0.05). Plots were generated using variations of DESeq2 plotting functions and dplyr, pheatmap, ggplot2, ggrepel, and ggbreak^108^ packages with R version 4.3.0. Annotation data from ENSEMBL 102 was used, and genes were additionally annotated with Entrez GeneIDs and text descriptions. Heatmaps and violin plots were generated from vst normalization values generated by DESeq2, and heatmaps were clustered by heatmap function using Euclidean distance and complete linkage. Public differentially expressed gene tables^36^^,^^59^ were compared to the differentially expressed genes found in our study. Differentially expressed genes from all three analyses were processed with Python to create a Pandas data frame with the Log2Fold change values of the common differentially expressed genes. The merged data frame was input into bioinfokit to generate the heatmaps. The Venn diagram was produced using VennDiagram based on the differentially expressed genes.

### Quantification and statistical analysis

Prism 10 software (GraphPad) was used for all statistical analysis. Unpaired t-test, one-way ANOVA, or two-way ANOVA were used for the statistical analysis of differences between two or more groups with *p* < 0.05 considered significant and indicated by asterisks: ∗∗∗∗*p* < 0.0001, ∗∗∗*p* < 0.001, ∗∗*p* < 0.01, ∗*p* < 0.05. ns for non-significant *p* values.

## References

[1] Eberl G., Colonna M., Di Santo J.P., McKenzie A.N.J. (2015). Innate lymphoid cells: a new paradigm in immunology. Science.

[2] Vivier E., Artis D., Colonna M., Diefenbach A., Di Santo J.P., Eberl G., Koyasu S., Locksley R.M., McKenzie A.N.J., Mebius R.E. (2018). Innate lymphoid cells: 10 years on. Cell.

[3] Seo G.Y., Giles D.A., Kronenberg M. (2020). The role of innate lymphoid cells in response to microbes at mucosal surfaces. Mucosal Immunol..

[4] Seillet C., Jacquelot N. (2019). Sensing of physiological regulators by innate lymphoid cells. Cell. Mol. Immunol..

[5] Sonnenberg G.F., Artis D. (2015). Innate lymphoid cells in the initiation, regulation and resolution of inflammation. Nat. Med..

[6] Quatrini L., Wieduwild E., Guia S., Bernat C., Glaichenhaus N., Vivier E., Ugolini S. (2017). Host resistance to endotoxic shock requires the neuroendocrine regulation of group 1 innate lymphoid cells. J. Exp. Med..

[7] Almeida F.F., Belz G.T. (2016). Innate lymphoid cells: models of plasticity for immune homeostasis and rapid responsiveness in protection. Mucosal Immunol..

[8] Bal S.M., Golebski K., Spits H. (2020). Plasticity of innate lymphoid cell subsets. Nat. Rev. Immunol..

[9] Silver J.S., Kearley J., Copenhaver A.M., Sanden C., Mori M., Yu L., Pritchard G.H., Berlin A.A., Hunter C.A., Bowler R. (2016). Inflammatory triggers associated with exacerbations of COPD orchestrate plasticity of group 2 innate lymphoid cells in the lungs. Nat. Immunol..

[10] Ohne Y., Silver J.S., Thompson-Snipes L., Collet M.A., Blanck J.P., Cantarel B.L., Copenhaver A.M., Humbles A.A., Liu Y.J. (2016). IL-1 is a critical regulator of group 2 innate lymphoid cell function and plasticity. Nat. Immunol..

[11] Cella M., Otero K., Colonna M. (2010). Expansion of human NK-22 cells with IL-7, IL-2, and IL-1beta reveals intrinsic functional plasticity. Proc. Natl. Acad. Sci. USA.

[12] Cella M., Gamini R., Sécca C., Collins P.L., Zhao S., Peng V., Robinette M.L., Schettini J., Zaitsev K., Gordon W. (2019). Subsets of ILC3-ILC1-like cells generate a diversity spectrum of innate lymphoid cells in human mucosal tissues. Nat. Immunol..

[13] Mazzurana L., Rao A., Van Acker A., Mjösberg J. (2018). The roles for innate lymphoid cells in the human immune system. Semin. Immunopathol..

[14] Crellin N.K., Trifari S., Kaplan C.D., Satoh-Takayama N., Di Santo J.P., Spits H. (2010). Regulation of cytokine secretion in human CD127(+) LTi-like innate lymphoid cells by Toll-like receptor 2. Immunity.

[15] Shih H.Y., Sciumè G., Mikami Y., Guo L., Sun H.W., Brooks S.R., Urban J.F., Davis F.P., Kanno Y., O'Shea J.J. (2016). Developmental Acquisition of Regulomes Underlies Innate Lymphoid Cell Functionality. Cell.

[16] Parker M.E., Barrera A., Wheaton J.D., Zuberbuehler M.K., Allan D.S.J., Carlyle J.R., Reddy T.E., Ciofani M. (2020). c-Maf regulates the plasticity of group 3 innate lymphoid cells by restraining the type 1 program. J. Exp. Med..

[17] Pokrovskii M., Hall J.A., Ochayon D.E., Yi R., Chaimowitz N.S., Seelamneni H., Carriero N., Watters A., Waggoner S.N., Littman D.R. (2019). Characterization of Transcriptional Regulatory Networks that Promote and Restrict Identities and Functions of Intestinal Innate Lymphoid Cells. Immunity.

[18] Cortez V.S., Cervantes-Barragan L., Robinette M.L., Bando J.K., Wang Y., Geiger T.L., Gilfillan S., Fuchs A., Vivier E., Sun J.C. (2016). Transforming growth factor-β signaling guides the differentiation of innate lymphoid cells in salivary glands. Immunity.

[19] Gao Y., Souza-Fonseca-Guimaraes F., Bald T., Ng S.S., Young A., Ngiow S.F., Rautela J., Straube J., Waddell N., Blake S.J. (2017). Tumor immunoevasion by the conversion of effector NK cells into type 1 innate lymphoid cells. Nat. Immunol..

[20] Cuff A.O., Sillito F., Dertschnig S., Hall A., Luong T.V., Chakraverty R., Male V. (2019). The Obese Liver Environment Mediates Conversion of NK Cells to a Less Cytotoxic ILC1-Like Phenotype. Front. Immunol..

[21] Cortez V.S., Ulland T.K., Cervantes-Barragan L., Bando J.K., Robinette M.L., Wang Q., White A.J., Gilfillan S., Cella M., Colonna M. (2017). SMAD4 impedes the conversion of NK cells into ILC1-like cells by curtailing non-canonical TGF-beta signaling. Nat. Immunol..

[22] Park E., Patel S., Wang Q., Andhey P., Zaitsev K., Porter S., Hershey M., Bern M., Plougastel-Douglas B., Collins P. (2019). Toxoplasma gondii infection drives conversion of NK cells into ILC1-like cells. Elife.

[23] Pikovskaya O., Chaix J., Rothman N.J., Collins A., Chen Y.H., Scipioni A.M., Vivier E., Reiner S.L. (2016). Cutting Edge: Eomesodermin Is Sufficient To Direct Type 1 Innate Lymphocyte Development into the Conventional NK Lineage. J. Immunol..

[24] Spits H., Bernink J.H., Lanier L. (2016). NK cells and type 1 innate lymphoid cells: partners in host defense. Nat. Immunol..

[25] Peng H., Jiang X., Chen Y., Sojka D.K., Wei H., Gao X., Sun R., Yokoyama W.M., Tian Z. (2013). Liver-resident NK cells confer adaptive immunity in skin-contact inflammation. J. Clin. Investig..

[26] Constantinides M.G., McDonald B.D., Verhoef P.A., Bendelac A. (2014). A committed precursor to innate lymphoid cells. Nature.

[27] O'Sullivan T.E., Rapp M., Fan X., Weizman O.E., Bhardwaj P., Adams N.M., Walzer T., Dannenberg A.J., Sun J.C. (2016). Adipose-Resident Group 1 Innate Lymphoid Cells Promote Obesity-Associated Insulin Resistance. Immunity.

[28] Dadi S., Chhangawala S., Whitlock B.M., Franklin R.A., Luo C.T., Oh S.A., Toure A., Pritykin Y., Huse M., Leslie C.S., Li M.O. (2016). Cancer immunosurveillance by tissue-resident innate lymphoid cells and innate-like T cells. Cell.

[29] Verma R., Er J.Z., Pu R.W., Sheik Mohamed J., Soo R.A., Muthiah H.M., Tam J.K.C., Ding J.L. (2020). Eomes expression defines group 1 innate lymphoid cells during metastasis in human and mouse. Front. Immunol..

[30] Knox J.J., Cosma G.L., Betts M.R., McLane L.M. (2014). Characterization of T-bet and eomes in peripheral human immune cells. Front. Immunol..

[31] Kotwica-Mojzych K., Jodłowska-Jędrych B., Mojzych M. (2021). CD200: CD200R interactions and their importance in immunoregulation. Int. J. Mol. Sci..

[32] Fathman J.W., Bhattacharya D., Inlay M.A., Seita J., Karsunky H., Weissman I.L. (2011). Identification of the earliest natural killer cell–committed progenitor in murine bone marrow. Blood.

[33] Meininger I., Carrasco A., Rao A., Soini T., Kokkinou E., Mjösberg J. (2020). Tissue-specific features of innate lymphoid cells. Trends Immunol..

[34] Smyth M.J., Cretney E., Takeda K., Wiltrout R.H., Sedger L.M., Kayagaki N., Yagita H., Okumura K. (2001). Tumor necrosis factor–related apoptosis-inducing ligand (TRAIL) contributes to interferon γ–dependent natural killer cell protection from tumor metastasis. J. Exp. Med..

[35] Kayagaki N., Yamaguchi N., Nakayama M., Takeda K., Akiba H., Tsutsui H., Okamura H., Nakanishi K., Okumura K., Yagita H. (1999). Expression and function of TNF-related apoptosis-inducing ligand on murine activated NK cells. J. Immunol..

[36] Weizman O.-E., Adams N.M., Schuster I.S., Krishna C., Pritykin Y., Lau C., Degli-Esposti M.A., Leslie C.S., Sun J.C., O’Sullivan T.E. (2017). ILC1 confer early host protection at initial sites of viral infection. Cell.

[37] Bezman N.A., Kim C.C., Sun J.C., Min-Oo G., Hendricks D.W., Kamimura Y., Best J.A., Goldrath A.W., Lanier L.L., Immunological Genome Project Consortium (2012). Molecular definition of the identity and activation of natural killer cells. Nat. Immunol..

[38] Arase H., Saito T., Phillips J.H., Lanier L.L. (2001). Cutting edge: the mouse NK cell-associated antigen recognized by DX5 moncoclonal antibody is CD49b (α2 integrin, very late antigen-2). J. Immunol..

[39] Vivier E., Tomasello E., Baratin M., Walzer T., Ugolini S. (2008). Functions of natural killer cells. Nat. Immunol..

[40] Sonnino S., Mauri L., Chigorno V., Prinetti A. (2007). Gangliosides as components of lipid membrane domains. Glycobiology.

[41] Rock P., Allietta M., Young W.W., Thompson T.E., Tillack T.W. (1990). Organization of glycosphingolipids in phosphatidylcholine bilayers: use of antibody molecules and Fab fragments as morphologic markers. Biochemistry.

[42] Kasai M., Iwamori M., Nagai Y., Okumura K., Tada T. (1980). A glycolipid on the surface of mouse natural killer cells. Eur. J. Immunol..

[43] Kasai M., Yoneda T., Habu S., Maruyama Y., Okumura K., Tokunaga T. (1981). In vivo effect of anti-asialo GM1 antibody on natural killer activity. Nature.

[44] Tang J., DeLong D.C., Marder P., Butler L.D., Ades E.W. (1985). Identification of functional subpopulations of murine natural killer cells based on their cell surface asialo GM1 phenotype. Cell. Immunol..

[45] Hargrove M.E., Ting C.-C. (1988). Asialo GM1 as an accessory molecule determining the function and reactivity of cytotoxic T lymphocytes. Cell. Immunol..

[46] Trambley J., Bingaman A.W., Lin A., Elwood E.T., Waitze S.Y., Ha J., Durham M.M., Corbascio M., Cowan S.R., Pearson T.C., Larsen C.P. (1999). Asialo GM1(+) CD8(+) T cells play a critical role in costimulation blockade-resistant allograft rejection. J. Clin. Investig..

[47] Yamanokuchi S., Ikai I., Nishitai R., Matsushita T., Sugimoto S., Shiotani T., Yamaoka Y. (2005). Asialo GM1 positive CD8+ T cells induce skin allograft rejection in the absence of the secondary lymphoid organs. J. Surg. Res..

[48] Zhang J., Le Gras S., Pouxvielh K., Faure F., Fallone L., Kern N., Moreews M., Mathieu A.L., Schneider R., Marliac Q. (2021). Sequential actions of EOMES and T-BET promote stepwise maturation of natural killer cells. Nat. Commun..

[49] Simonetta F., Pradier A., Roosnek E. (2016). T-bet and eomesodermin in NK cell development, maturation, and function. Front. Immunol..

[50] Kim S., Iizuka K., Kang H.S.P., Dokun A., French A.R., Greco S., Yokoyama W.M. (2002). In vivo developmental stages in murine natural killer cell maturation. Nat. Immunol..

[51] Millan A.J., Hom B.A., Libang J.B., Sindi S., Manilay J.O. (2021). Evidence for Prescribed NK Cell Ly-49 Developmental Pathways in Mice. J. Immunol..

[52] Abel A.M., Yang C., Thakar M.S., Malarkannan S. (2018). Natural Killer Cells: Development, Maturation, and Clinical Utilization. Front. Immunol..

[53] Schorr C., Krishnan M.S., Capitano M. (2023). Deficits in our understanding of natural killer cell development in mouse and human. Curr. Opin. Hematol..

[54] Klose C.S.N., Flach M., Möhle L., Rogell L., Hoyler T., Ebert K., Fabiunke C., Pfeifer D., Sexl V., Fonseca-Pereira D. (2014). Differentiation of type 1 ILCs from a common progenitor to all helper-like innate lymphoid cell lineages. Cell.

[55] Constantinides M.G., Gudjonson H., McDonald B.D., Ishizuka I.E., Verhoef P.A., Dinner A.R., Bendelac A. (2015). PLZF expression maps the early stages of ILC1 lineage development. Proc. Natl. Acad. Sci. USA.

[56] Shinkai Y., Rathbun G., Lam K.P., Oltz E.M., Stewart V., Mendelsohn M., Charron J., Datta M., Young F., Stall A.M. (1992). RAG-2-deficient mice lack mature lymphocytes owing to inability to initiate V (D) J rearrangement. Cell.

[57] Friedrich C., Taggenbrock R.L.R.E., Doucet-Ladevèze R., Golda G., Moenius R., Arampatzi P., Kragten N.A.M., Kreymborg K., Gomez de Agüero M., Kastenmüller W. (2021). Effector differentiation downstream of lineage commitment in ILC1s is driven by Hobit across tissues. Nat. Immunol..

[58] Lopes N., Galluso J., Escaliere B., Carpentier S., Kerdiles Y.M., Vivier E. (2022). Tissue-specific transcriptional profiles and heterogeneity of natural killer cells and group 1 innate lymphoid cells. Cell Rep. Med..

[59] Robinette M.L., Fuchs A., Cortez V.S., Lee J.S., Wang Y., Durum S.K., Gilfillan S., Colonna M., Immunological Genome Consortium (2015). Transcriptional programs define molecular characteristics of innate lymphoid cell classes and subsets. Nat. Immunol..

[60] Elpek K.G., Cremasco V., Shen H., Harvey C.J., Wucherpfennig K.W., Goldstein D.R., Monach P.A., Turley S.J. (2014). The tumor microenvironment shapes lineage, transcriptional, and functional diversity of infiltrating myeloid cells. Cancer Immunol. Res..

[61] Luci C., Bihl F., Bourdely P., Khou S., Popa A., Meghraoui-Kheddar A., Vermeulen O., Elaldi R., Poissonnet G., Sudaka A. (2021). Cutaneous Squamous Cell Carcinoma Development Is Associated with a Temporal Infiltration of ILC1 and NK Cells with Immune Dysfunctions. J. Invest. Dermatol..

[62] Xiong J., Zhao Y., Lin Y., Chen L., Weng Q., Shi C., Liu X., Geng Y., Liu L., Wang J., Zhang M. (2022). Identification and characterization of innate lymphoid cells generated from pluripotent stem cells. Cell Rep..

[63] Flommersfeld S., Bottcher J.P., Ersching J., Flossdorf M., Meiser P., Pachmayr L.O., Leube J., Hensel I., Jarosch S., Zhang Q. (2021). Fate mapping of single NK cells identifies a type 1 innate lymphoid-like lineage that bridges innate and adaptive recognition of viral infection. Immunity.

[64] Dai G., Sun Y., Wei R., Xi L. (2023). Small Leucine-Rich Proteoglycan PODNL1 Identified as a Potential Tumor Matrix-Mediated Biomarker for Prognosis and Immunotherapy in a Pan-Cancer Setting. Curr. Issues Mol. Biol..

[65] Hugonnet M., Singh P., Haas Q., von Gunten S. (2021). The Distinct Roles of Sialyltransferases in Cancer Biology and Onco-Immunology. Front. Immunol..

[66] Kamata K., Mizutani K., Takahashi K., Marchetti R., Silipo A., Addy C., Park S.Y., Fujii Y., Fujita H., Konuma T. (2020). The structure of SeviL, a GM1b/asialo-GM1 binding R-type lectin from the mussel Mytilisepta virgata. Sci. Rep..

[67] Martinet L., Ferrari De Andrade L., Guillerey C., Lee J.S., Liu J., Souza-Fonseca-Guimaraes F., Hutchinson D.S., Kolesnik T.B., Nicholson S.E., Huntington N.D., Smyth M.J. (2015). DNAM-1 expression marks an alternative program of NK cell maturation. Cell Rep..

[68] Huntington N.D., Martinet L., Smyth M.J. (2015). DNAM-1: would the real natural killer cell please stand up. Oncotarget.

[69] Kaiserman D., Bird P.I. (2010). Control of granzymes by serpins. Cell Death Differ..

[70] Pachynski R.K., Zabel B.A., Kohrt H.E., Tejeda N.M., Monnier J., Swanson C.D., Holzer A.K., Gentles A.J., Sperinde G.V., Edalati A. (2012). The chemoattractant chemerin suppresses melanoma by recruiting natural killer cell antitumor defenses. J. Exp. Med..

[71] Lachota M., Zielniok K., Palacios D., Kanaya M., Penna L., Hoel H.J., Wiiger M.T., Kveberg L., Hautz W., Zagożdżon R., Malmberg K.J. (2023). Mapping the chemotactic landscape in NK cells reveals subset-specific synergistic migratory responses to dual chemokine receptor ligation. EBioMedicine.

[72] Ballet R., LaJevic M., Huskey-Mullin N., Roach R., Brulois K., Huang Y., Saeed M.A., Dang H.X., Pachynski R.K., Wilson E. (2023). Chemerin triggers migration of a CD8 T cell subset with natural killer cell functions. Mol. Ther..

[73] Okamura H., Tsutsi H., Komatsu T., Yutsudo M., Hakura A., Tanimoto T., Torigoe K., Okura T., Nukada Y., Hattori K. (1995). Cloning of a new cytokine that induces IFN-γ production by T cells. Nature.

[74] Nandagopal N., Ali A.K., Komal A.K., Lee S.-H. (2014). The critical role of IL-15–PI3K–mTOR pathway in natural killer cell effector functions. Front. Immunol..

[75] Gill S., Vasey A.E., De Souza A., Baker J., Smith A.T., Kohrt H.E., Florek M., Gibbs K.D., Tate K., Ritchie D.S., Negrin R.S. (2012). Rapid development of exhaustion and down-regulation of eomesodermin limit the antitumor activity of adoptively transferred murine natural killer cells. Blood.

[76] Nabekura T., Riggan L., Hildreth A.D., O’Sullivan T.E., Shibuya A. (2020). Type 1 innate lymphoid cells protect mice from acute liver injury via interferon-γ secretion for upregulating Bcl-xL expression in hepatocytes. Immunity.

[77] Rothman J., Jarriault S. (2019). Developmental Plasticity and Cellular Reprogramming in Caenorhabditis elegans. Genetics.

[78] Laouar Y., Sutterwala F.S., Gorelik L., Flavell R.A. (2005). Transforming growth factor-β controls T helper type 1 cell development through regulation of natural killer cell interferon-γ. Nat. Immunol..

[79] Marcoe J.P., Lim J.R., Schaubert K.L., Fodil-Cornu N., Matka M., McCubbrey A.L., Farr A.R., Vidal S.M., Laouar Y. (2012). TGF-beta is responsible for NK cell immaturity during ontogeny and increased susceptibility to infection during mouse infancy. Nat. Immunol..

[80] Li X., Li C., Zhang W., Wang Y., Qian P., Huang H. (2023). Inflammation and aging: signaling pathways and intervention therapies. Signal Transduct. Target. Ther..

[81] Spits H., Artis D., Colonna M., Diefenbach A., Di Santo J.P., Eberl G., Koyasu S., Locksley R.M., McKenzie A.N.J., Mebius R.E. (2013). Innate lymphoid cells—a proposal for uniform nomenclature. Nat. Rev. Immunol..

[82] Kabacik S., Lowe D., Fransen L., Leonard M., Ang S.L., Whiteman C., Corsi S., Cohen H., Felton S., Bali R. (2022). The relationship between epigenetic age and the hallmarks of aging in human cells. Nat. Aging.

[83] Franceschi C., Garagnani P., Vitale G., Capri M., Salvioli S. (2017). Inflammaging and Garb-aging. Trends Endocrinol. Metab..

[84] Fauriat C., Just-Landi S., Mallet F., Arnoulet C., Sainty D., Olive D., Costello R.T. (2007). Deficient expression of NCR in NK cells from acute myeloid leukemia: Evolution during leukemia treatment and impact of leukemia cells in NCRdull phenotype induction. Blood.

[85] Costello R.T., Sivori S., Marcenaro E., Lafage-Pochitaloff M., Mozziconacci M.J., Reviron D., Gastaut J.A., Pende D., Olive D., Moretta A. (2002). Defective expression and function of natural killer cell-triggering receptors in patients with acute myeloid leukemia. Blood.

[86] Mamessier E., Sylvain A., Thibult M.L., Houvenaeghel G., Jacquemier J., Castellano R., Gonçalves A., André P., Romagné F., Thibault G. (2011). Human breast cancer cells enhance self tolerance by promoting evasion from NK cell antitumor immunity. J. Clin. Investig..

[87] Platonova S., Cherfils-Vicini J., Damotte D., Crozet L., Vieillard V., Validire P., André P., Dieu-Nosjean M.C., Alifano M., Régnard J.F. (2011). Profound coordinated alterations of intratumoral NK cell phenotype and function in lung carcinoma. Cancer Res..

[88] Gillard-Bocquet M., Caer C., Cagnard N., Crozet L., Perez M., Fridman W.H., Sautès-Fridman C., Cremer I. (2013). Lung tumor microenvironment induces specific gene expression signature in intratumoral NK cells. Front. Immunol..

[89] Pietra G., Manzini C., Rivara S., Vitale M., Cantoni C., Petretto A., Balsamo M., Conte R., Benelli R., Minghelli S. (2012). Melanoma cells inhibit natural killer cell function by modulating the expression of activating receptors and cytolytic activity. Cancer Res..

[90] Konjevic G., Mirjacic Martinovic K., Vuletic A., Jovic V., Jurisic V., Babovic N., Spuzic I. (2007). Low expression of CD161 and NKG2D activating NK receptor is associated with impaired NK cell cytotoxicity in metastatic melanoma patients. Clin. Exp. Metastasis.

[91] Nieto-Velazquez N.G., Torres-Ramos Y.D., Munoz-Sanchez J.L., Espinosa-Godoy L., Gomez-Cortes S., Moreno J., Moreno-Eutimio M.A. (2016). Altered Expression of Natural Cytotoxicity Receptors and NKG2D on Peripheral Blood NK Cell Subsets in Breast Cancer Patients. Transl. Oncol..

[92] Jang Y., Gerbec Z.J., Won T., Choi B., Podsiad A., B Moore B., Malarkannan S., Laouar Y. (2018). Cutting edge: check your mice—a point mutation in the Ncr1 locus identified in CD45. 1 congenic mice with consequences in mouse susceptibility to infection. J. Immunol..

[93] Almeida F.F., Tognarelli S., Marçais A., Kueh A.J., Friede M.E., Liao Y., Willis S.N., Luong K., Faure F., Mercier F.E. (2018). A point mutation in the Ncr1 signal peptide impairs the development of innate lymphoid cell subsets. OncoImmunology.

[94] Maccioni H.J., Daniotti J.L., Martina J.A. (1999). Organization of ganglioside synthesis in the Golgi apparatus. Biochim. Biophys. Acta.

[95] Robert K.Y., Bieberich E., Xia T., Zeng G. (2004). Regulation of ganglioside biosynthesis in the nervous system. J. Lipid Res..

[96] Yu J., Freud A.G., Caligiuri M.A. (2013). Location and cellular stages of natural killer cell development. Trends Immunol..

[97] Moreno Nieves U.Y., Tay J., Saumyaa S., Mundy D., Sunwoo J.B. (2020). Plasticity and Polarization of Human NK Cells in the Tumor Microenvironment. J. Immunol..

[98] Dustin M.L., Long E.O. (2010). Cytotoxic immunological synapses. Immunol. Rev..

[99] Das A., Long E.O. (2010). Lytic granule polarization, rather than degranulation, is the preferred target of inhibitory receptors in NK cells. J. Immunol..

[100] Maul-Pavicic A., Chiang S.C.C., Rensing-Ehl A., Jessen B., Fauriat C., Wood S.M., Sjöqvist S., Hufnagel M., Schulze I., Bass T. (2011). ORAI1-mediated calcium influx is required for human cytotoxic lymphocyte degranulation and target cell lysis. Proc. Natl. Acad. Sci. USA.

[101] Nixon B.G., Chou C., Krishna C., Dadi S., Michel A.O., Cornish A.E., Kansler E.R., Do M.H., Wang X., Capistrano K.J. (2022). Cytotoxic granzyme C-expressing ILC1s contribute to antitumor immunity and neonatal autoimmunity. Sci. Immunol..

[102] Kannan G., Thaprawat P., Schultz T.L., Carruthers V.B. (2021). Acquisition of host cytosolic protein by Toxoplasma gondii Bradyzoites. mSphere.

[103] Martin M. (2011). Cutadapt removes adapter sequences from high-throughput sequencing reads. EMBnet. J..

[104] Wingett S.W., Andrews S. (2018). FastQ Screen: A tool for multi-genome mapping and quality control. F1000Res..

[105] Dobin A., Davis C.A., Schlesinger F., Drenkow J., Zaleski C., Jha S., Batut P., Chaisson M., Gingeras T.R. (2013). STAR: ultrafast universal RNA-seq aligner. Bioinformatics.

[106] Li B., Dewey C.N. (2011). RSEM: accurate transcript quantification from RNA-Seq data with or without a reference genome. BMC Bioinf..

[107] Ewels P., Magnusson M., Lundin S., Käller M. (2016). MultiQC: summarize analysis results for multiple tools and samples in a single report. Bioinformatics.

[108] Xu S., Chen M., Feng T., Zhan L., Zhou L., Yu G. (2021). Use ggbreak to effectively utilize plotting space to deal with large datasets and outliers. Front. Genet..

[109] Love M.I., Huber W., Anders S. (2014). Moderated estimation of fold change and dispersion for RNA-seq data with DESeq2. Genome Biol..

