# Supplementary material for: A membrane lipid signature unravels the dynamic landscape of group 1 innate lymphoid cells across the health-disease continuum
Source: iScience. 2025 Feb 17;28(3):112043. doi: 10.1016/j.isci.2025.112043 (PMC11914809; doi:10.1016/j.isci.2025.112043)
Supplement: Document S1. Figures S1–S8 [file mmc1.pdf]

iScience, Volume *28*

Supplemental information

A membrane lipid signature unravels the dynamic landscape of group 1 innate lymphoid cells across the health-disease continuum

Halle C. Frey, Xin Sun, Fatima Oudeif, Darleny L. Corona, Zijun He, Taejoon Won, Tracy L. Schultz, Vern B. Carruthers, Amale Laouar, and Yasmina Laouar

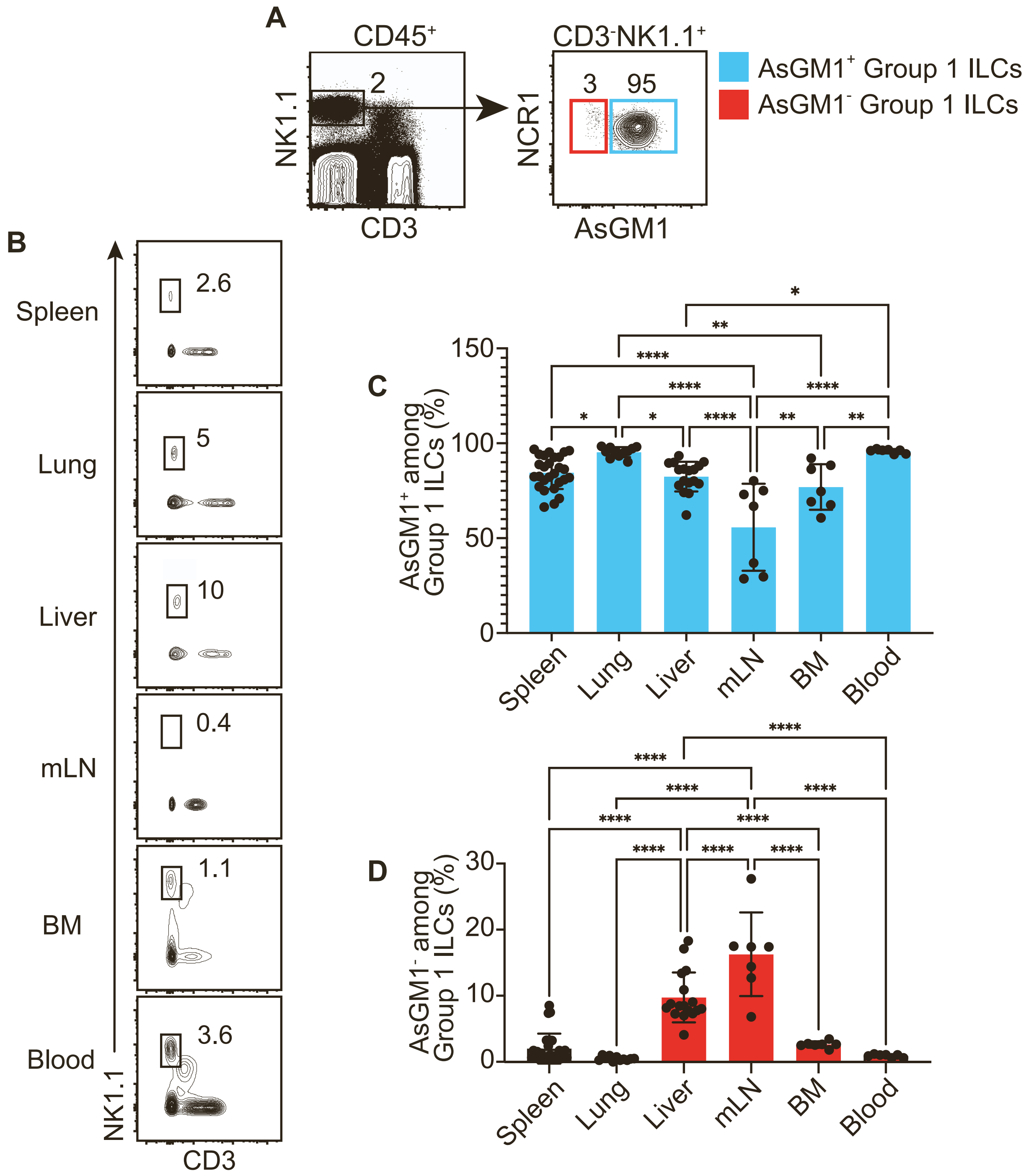

Figure S1. Distribution of AsGM1⁻ and AsGM1⁺ in Group 1 ILCs across tissues, Related to Figure 1. (A) Gating strategy for identifying AsGM1⁻ and AsGM1⁺ Group1 ILCs. (B) Distribution of NK1.1 vs CD3 in the spleen, lung, liver, mesenteric lymph nodes (mLN), bone marrow (BM), and blood. (C, D) Frequency of AsGM1⁺ (C) and AsGM1⁻ (D) cells among Group 1 ILCs (CD3⁻NK1.1⁺NCR1⁺) across tissues. Data represents 3 independent experiments: spleen (n = 27), lung (n = 5), liver (n = 16), mLN (n = 7), BM (n = 7), and blood (n = 7). Statistical analysis was performed using one-way ANOVA (C, D), with significance indicated by *p<0.05, **p<0.01, ****p<0.0001. Error bars show mean + s.d.

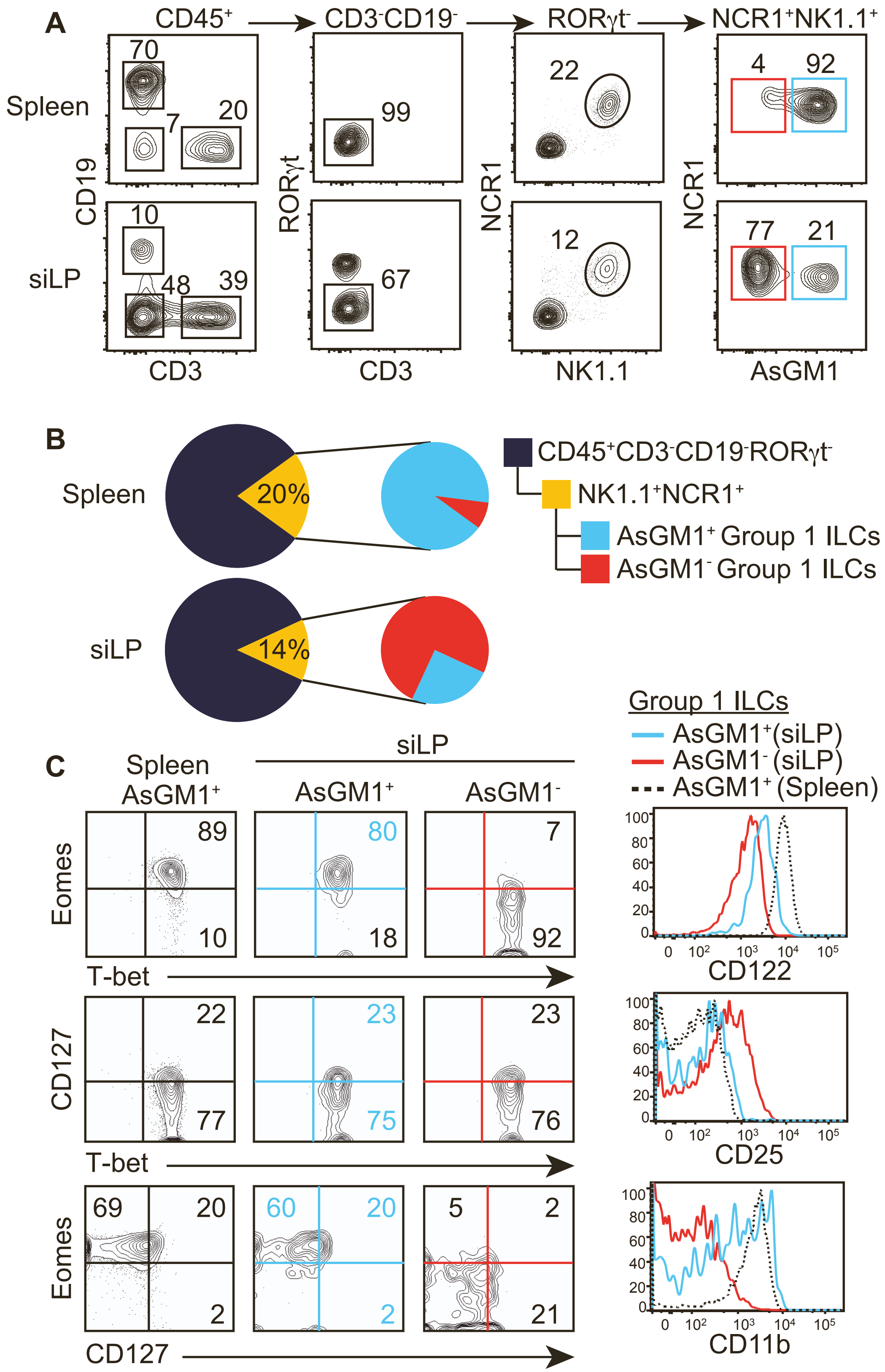

Figure S2. AsGM1 segregates intestinal Group 1 ILCs into ILC1 and NK cell subsets, Related to Figure 1. (A) Gating strategy for identifying AsGM1$^-$ and AsGM1$^+$ Group1 ILCs. (B) Ratio of AsGM1$^-$ (red) to AsGM1$^+$ (blue) cells within Group 1 ILCs (yellow) in spleen and small intestine lamina propria (siLP). (C) Expression of Eomes, T-bet, CD127, CD122, CD25, and CD11b in intestinal AsGM1$^-$ and AsGM1$^+$ Group 1 ILCs. Splenic AsGM1$^+$ Group 1 ILCs serve as reference. Data represents 2 independent experiments with n = 6.

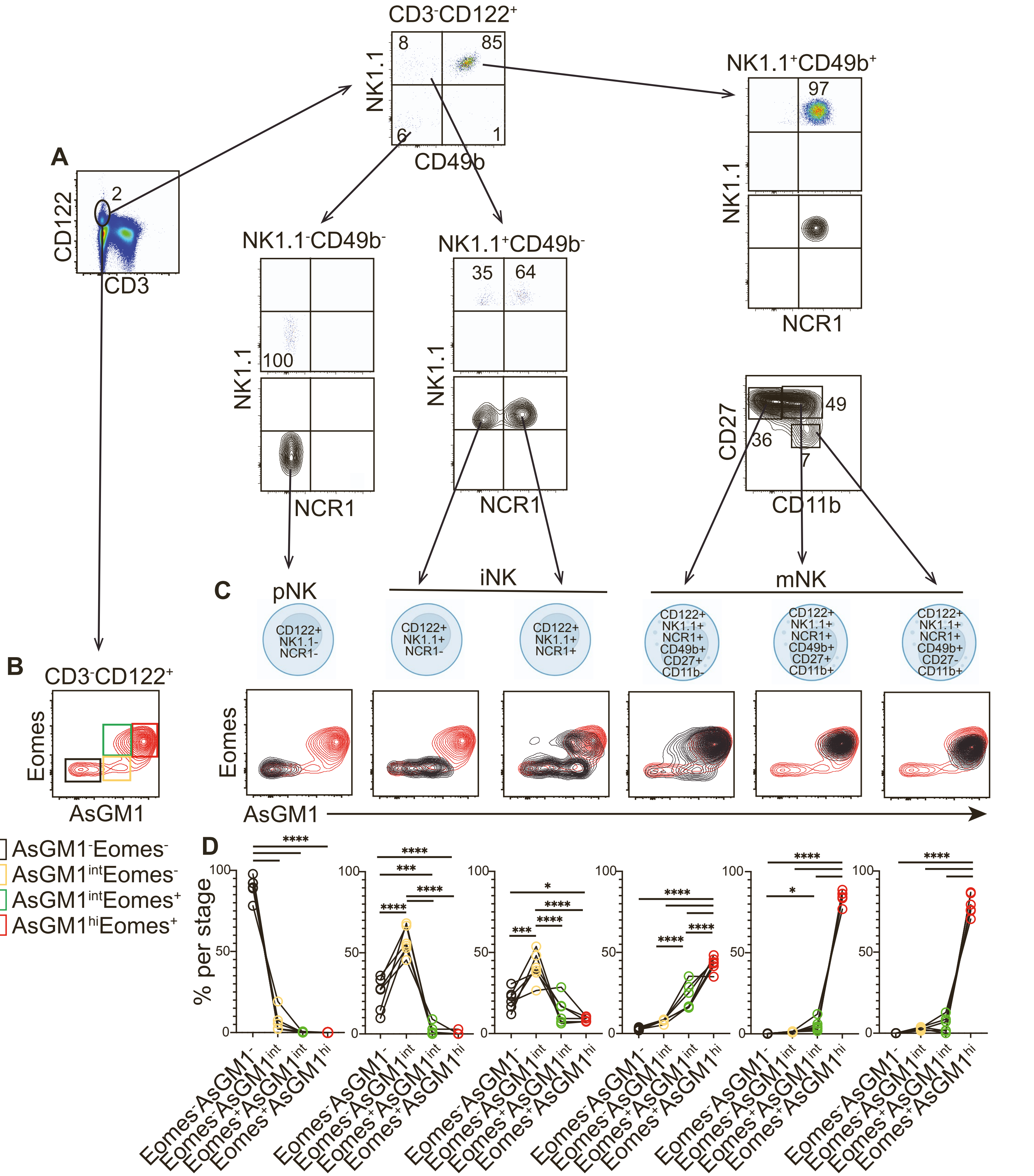

Figure S3. AsGM1 expression precedes Eomes during NK cell development, Related Figure 2. (A) Gating strategy delineating the progression from pNK to iNK and mNK cells within the bone marrow. (B) Distribution of Eomes versus AsGM1 expression across the total $CD122^{+}CD3^{-}$ cell compartment, with color-coded gates identifying four subsets: $AsGM1^{-}Eomes^{-}$, $AsGM1^{int}Eomes^{-}$, $AsGM1^{int}Eomes^{+}$, and $AsGM1^{hi}Eomes^{+}$. (C) Comparative distribution of Eomes and AsGM1 among specified developmental stages, with overlays highlighting cell subsets at each stage (navy) against the backdrop of the total $CD3^{-}CD122^{+}$ cell compartment (red). (D) Proportions of $AsGM1^{-}Eomes^{-}$ (black), $AsGM1^{int}Eomes^{-}$ (yellow), $AsGM1^{int}Eomes^{+}$ (green), and $AsGM1^{hi}Eomes^{+}$ (red) within each indicated developmental stage. Data represents 2 independent experiments with n = 6 mice. Statistical analysis was performed using one-way ANOVA, with significance indicated by *$p<0.05$, **$p<0.01$, ***$p<0.001$, ****$p<0.0001$. Individual mice are shown.

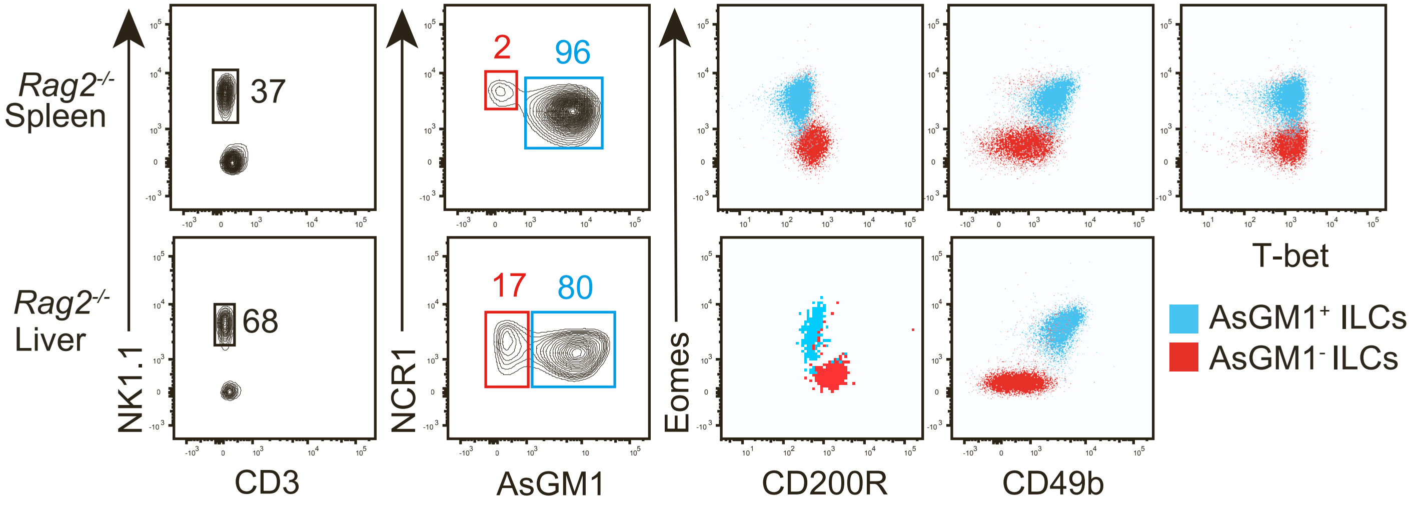

Figure S4. AsGM1- and AsGM1+ Group 1 ILCs from *Rag2*$^{-/-}$ mice used as input for RNA-seq analysis, Related to Figure 3. NK1.1 vs. CD3 distribution in the spleen and liver of *Rag2*$^{-/-}$ mice. NCR1 vs. AsGM1 distribution among CD3^{-}NK1.1^{+} ILCs. Distribution of Eomes vs. CD200R, CD49b, and T-bet among AsGM1- (red) and AsGM1+ (blue) Group 1 ILCs. Data represent 3 independent experiments: spleen (n = 6) and liver (n = 4).

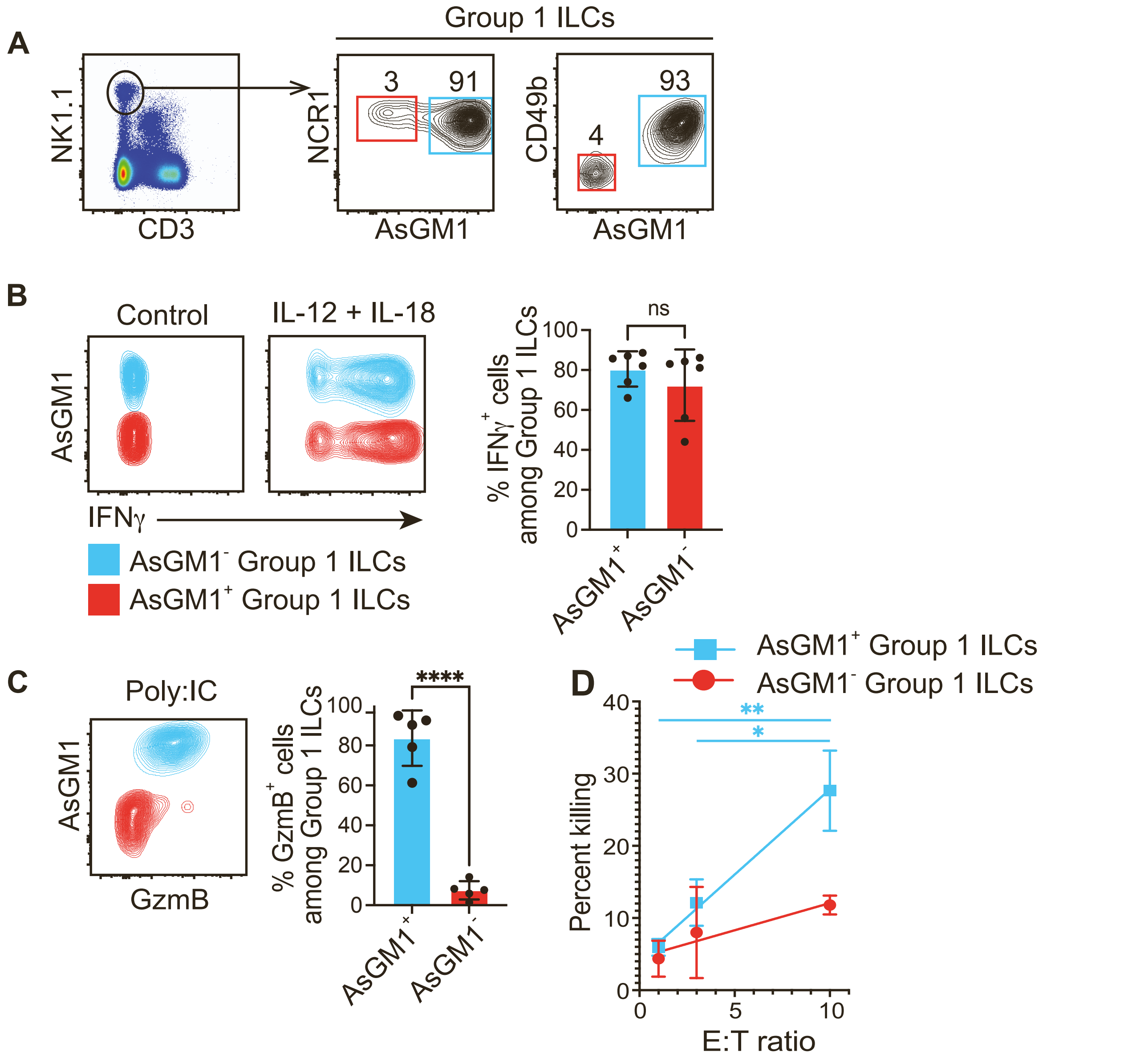

Figure S5. Splenic AsGM1$^+$ and AsGM1$^-$ Group 1 ILCs share IFNγ expression but exhibit differential cytotoxicity, Related to Figure 4. (A) Gating strategy for identifying AsGM1$^+$ (CD3$^-$NK1.1$^+$NCR1$^+$CD49b$^+$) and AsGM1$^-$ (CD3$^-$NK1.1$^+$NCR1$^+$CD49b$^-$) Group 1 ILCs. (B) Cells either remained untreated or were stimulated with 5 ng/ml IL-12 and 25 ng/ml IL-18 for 5 hours, with GolgiStop added after 4 hours. Panels show distribution and frequency of IFNγ among gated AsGM1$^-$ and AsGM1+ Group 1 ILCs. (C) Adult C57Bl/6 mice were treated (i.p.) with 100mg per mouse of Poly:IC (Sigma) for 16 hours. Panels show distribution and frequency of Granzym B (GzmB) expression among gated AsGM1$^-$ and AsGM1$^+$ Group 1 ILCs. (D) Sorted AsGM1$^-$ and AsGM1$^+$ Group 1 ILCs were cultured with CFSE-labeled YAC-1 cells at effector to target ratios of 1:1, 3:1, and 10:1. After 4 hours, cells were stained with live/dead marker (Invitrogen) and analyzed by flow cytometry. Percent lysis was calculated using the formula: [(% of dead CFSE$^+$ cells among target cells with effector) − (% of dead CFSE$^+$ cells among target cells without effector) / 100 - (% of dead CFSE$^+$ cells among target cells without effector)] /100. Data represent 3 (A-B) and 2 (C-D) independent experiments with n = 5-6 (A-C) and n = 2-5 (D). Statistical analysis was performed using an unpaired t-test (B, C) or two-way ANOVA (D) with significance indicated by *$p<0.05$, **$p<0.01$, ****$p<0.0001$. Error bars show mean + s.d (B, C) or s.e.m. (D). Nonlinear fitting was employed in (D).

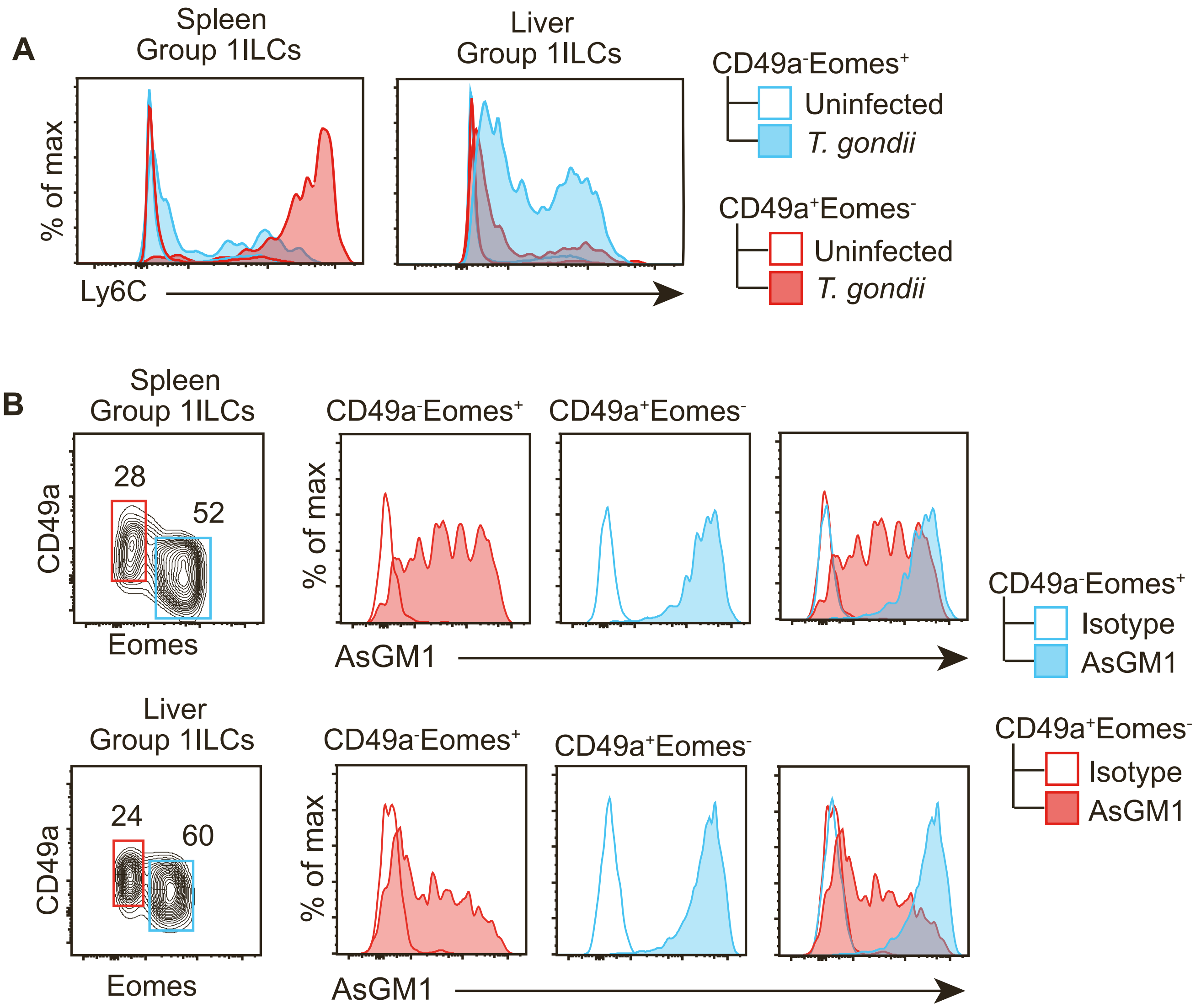

Figure S6. Alterations within the Group 1 ILC compartment driven by Toxoplasma gondii infection, Related to Figure 6. Expression of Ly6C (A) and AsGM1 (B) in ILC1s (CD49a$^+$Eomes$^-$) and NK cells (CD49a$^-$Eomes$^+$) from the spleen and liver (A, B) of uninfected (A) or T. gondii infected (A, B) mice 14 days post-infection. Data represent 2 independent experiments, with n = 6-11 uninfected mice and n = 11-16 infected mice. Representative isotype control (Rabbit IgG isotype control, ThermoFisher) from n = 2.

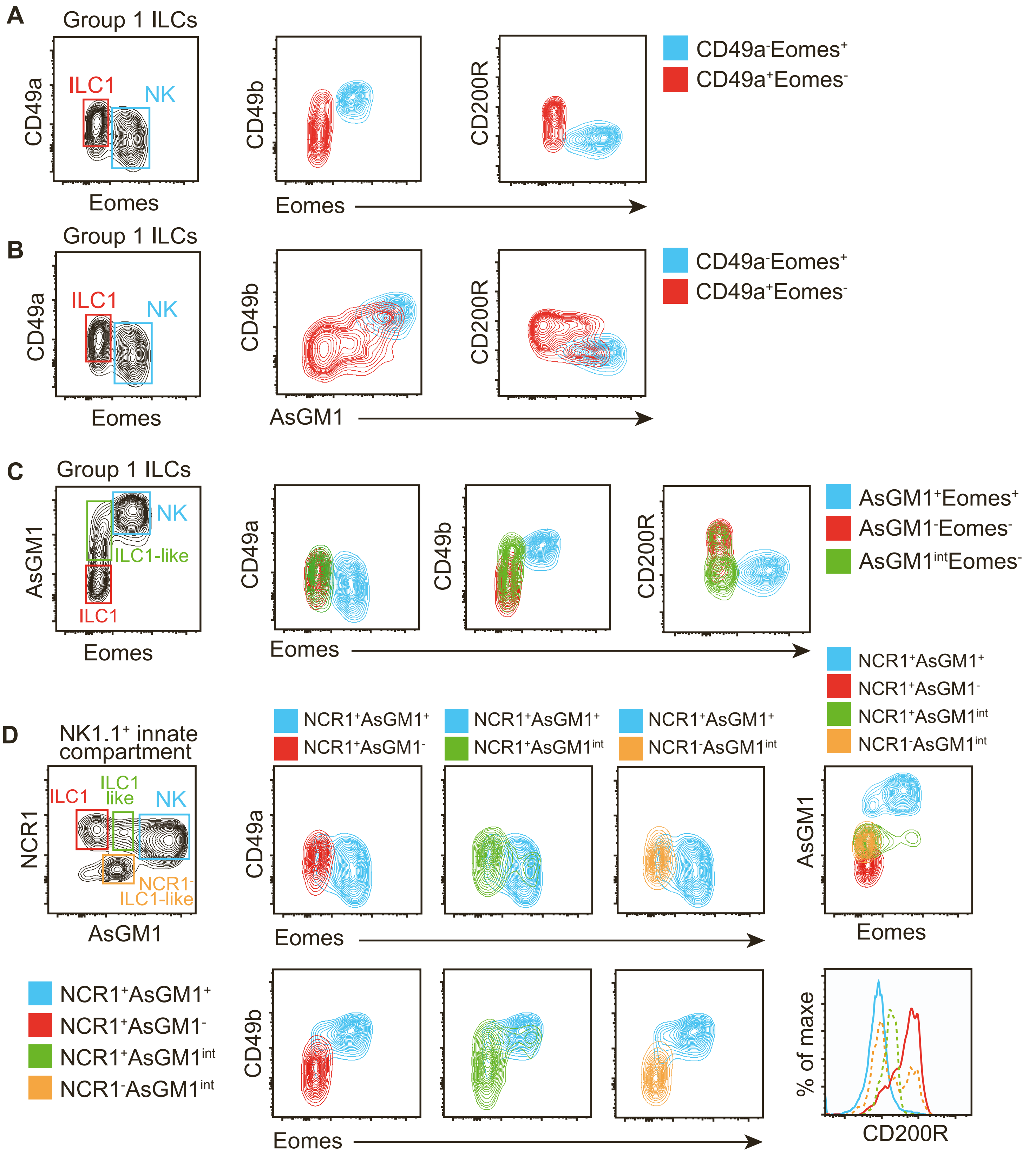

Figure S7. AsGM1 gradient unveils finite subsets within the ILC1 compartment during *T. gondii* infection, Related to Figure 6. (A-C) Group 1 ILCs (CD45$^+$CD3$^-$NK1.1$^+$NCR1$^+$) and (D) NK1.1$^+$ innate cell compartment (CD45$^+$CD3$^-$NK1.1$^+$) in the liver from *T. gondii* infected mice 14 days post-infection. (A) CD49b and CD200R among CD49a$^+$Eomes$^-$ and CD49a$^-$Eomes$^+$ subsets. (B) CD49b, CD200R and AsGM1 among CD49a$^+$Eomes$^-$ and CD49a$^-$Eomes$^+$ subsets. (C) CD49a, CD49b, and CD200R among AsGM1-segregated cells (Eomes$^+$AsGM1$^+$, Eomes$^-$AsGM1$^-$, and Eomes$^-$AsGM1^{int}). (D) CD49a, CD49b, Eomes, and CD200R among AsGM1-segregated cell subsets (NCR1$^+$AsGM1$^+$, NCR1$^+$AsGM1$^-$, NCR1$^+$AsGM1^{int}, and NCR1$^-$AsGM1^{int}). Data represent 2 independent experiments with n = 6-11 uninfected mice and n = 11-16 infected mice.

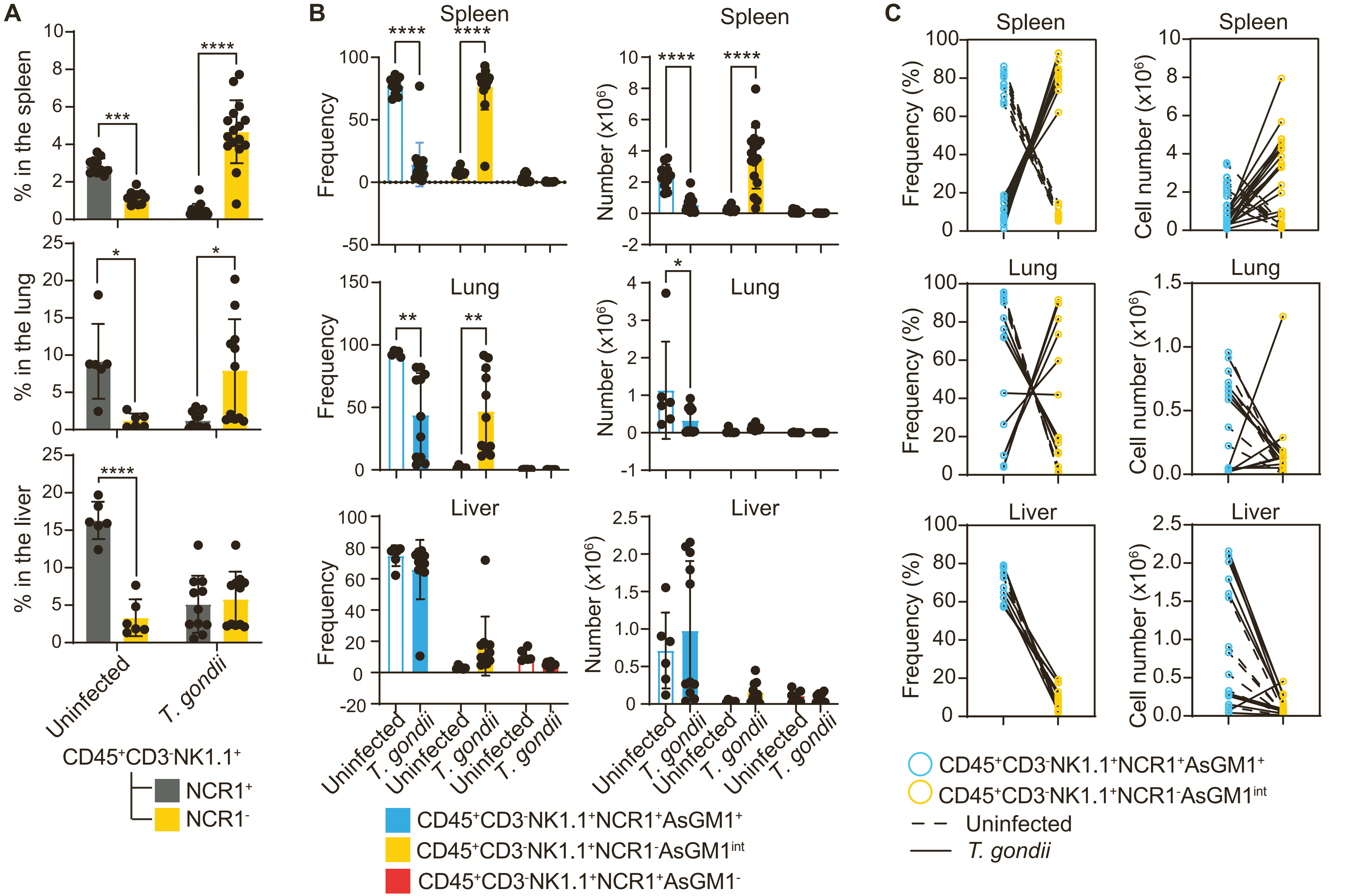

Figure S8. Greater diversity within Group 1 ILCs driven by *T. gondii* infection, Related to Figure 6. (A) Frequency of $NCR1^+$ and $NCR1^-$ innate lymphoid cells in the spleen, lung, and liver of uninfected or *T. gondii* infected mice 14 days post-infection. (B) Frequency of NK cells ($AsGM1^+NCR1^+$; blue), $NCR1^-AsGM1^{int}$ ILC1-like cells ($AsGM1^{int}NCR1^-$; yellow), and ILC1s ($AsGM1^-NCR1^+$; red) among $NK1.1^+$ innate cells in the spleen, lung, and liver of uninfected or T. gondii infected mice 14 days post-infection. (C) Correlation between frequency and number of NK cells vs. $NCR1^-AsGM1^{int}$ ILCs in the spleen, lung, and liver of uninfected and *T. gondii* infected mice 14 days post infection. Each line represents an individual mouse. Data represent 2 independent experiments, with n = 6-11 uninfected mice and n = 11-16 infected mice. Statistical analysis was performed using two-way ANOVA, with significance indicated by *p<0.05, **p<0.01, ****p<0.0001. Error bars show mean + s.d.
